# Protein 4.1B Contributes to the Organization of Peripheral Myelinated Axons

**DOI:** 10.1371/journal.pone.0025043

**Published:** 2011-09-26

**Authors:** Carmen Cifuentes-Diaz, Fabrice Chareyre, Marta Garcia, Jérôme Devaux, Michèle Carnaud, Grégoire Levasseur, Michiko Niwa-Kawakita, Sheila Harroch, Jean-Antoine Girault, Marco Giovannini, Laurence Goutebroze

**Affiliations:** 1 Inserm, UMR-S 839, Paris, France; 2 Université Pierre et Marie Curie (UPMC), Paris, France; 3 Institut du Fer à Moulin, Paris, France; 4 Inserm, U674, Institut Universitaire d'Hématologie, Paris, France; 5 Département de Signalisation Neuronale, CRN2M, UMR 6231, CNRS, Université de la Méditerranée-Université Paul Cézanne, IFR Jean Roche, Marseille, France; 6 Département de Neuroscience, Institut Pasteur, Paris, France; Institut de la Vision, France

## Abstract

Neurons are characterized by extremely long axons. This exceptional cell shape is likely to depend on multiple factors including interactions between the cytoskeleton and membrane proteins. In many cell types, members of the protein 4.1 family play an important role in tethering the cortical actin-spectrin cytoskeleton to the plasma membrane. Protein 4.1B is localized in myelinated axons, enriched in paranodal and juxtaparanodal regions, and also all along the internodes, but not at nodes of Ranvier where are localized the voltage-dependent sodium channels responsible for action potential propagation. To shed light on the role of protein 4.1B in the general organization of myelinated peripheral axons, we studied 4.1B knockout mice. These mice displayed a mildly impaired gait and motility. Whereas nodes were unaffected, the distribution of Caspr/paranodin, which anchors 4.1B to the membrane, was disorganized in paranodal regions and its levels were decreased. In juxtaparanodes, the enrichment of Caspr2, which also interacts with 4.1B, and of the associated TAG-1 and Kv1.1, was absent in mutant mice, whereas their levels were unaltered. Ultrastructural abnormalities were observed both at paranodes and juxtaparanodes. Axon calibers were slightly diminished in phrenic nerves and preterminal motor axons were dysmorphic in skeletal muscle. βII spectrin enrichment was decreased along the axolemma. Electrophysiological recordings at 3 post-natal weeks showed the occurrence of spontaneous and evoked repetitive activity indicating neuronal hyperexcitability, without change in conduction velocity. Thus, our results show that in myelinated axons 4.1B contributes to the stabilization of membrane proteins at paranodes, to the clustering of juxtaparanodal proteins, and to the regulation of the internodal axon caliber.

## Introduction

A distinctive property of neurons is their extremely elongated shape with an elaborate dendritic tree and axons that can be a meter long, or more. The caliber of the axon is maintained constant over long distances between bifurcations, with the exception of regular changes at the level of nodes of Ranvier in myelinated fibers. This regularity implies the existence of precise molecular interactions between the membrane and the cortical cytoskeleton which result in a regular curvature and a constant diameter. In many cell types the actin-spectrin cytoskeleton interacts with specific transmembrane proteins through linker proteins, making up a highly ramified cortical network. One of the major families of proteins involved in the interactions between the cytoskeleton and the membrane are the band 4.1 proteins (for review see [1]). Protein 4.1R, the first identified member of the family, is a key protein in red blood cells, where it anchors the actin-spectrin cytoskeleton to glycophorin C. This anchoring is critical for the biconcave shape of red blood cells since mutations in either protein 4.1R or glycophorin C result in hereditary elliptocytosis and hemolytic anemia. The family encompasses five members coded by different genes (4.1R, 4.1B, 4.1N, 4.1G and 4.1O), which can undergo alternative splicing [1], [2]. 4.1B (also termed type II brain 4.1, KIAA0987, EPB41L3) is highly enriched in the brain [3], [4]. A truncated form of 4.1B termed DAL-1 is a tumor suppressor involved in the pathogenesis of non-small cell lung and colorectal carcinomas, meningiomas, ependynomas, and breast and prostate cancers [5]–[11].

The myelinated axons are organized in specialized domains ensuring the rapid propagation of action potentials and are characterized by the presence of specific proteins underlying axoglial interactions (for review see [12], [13]). Voltage-gated sodium (Na_v_) channels are concentrated at nodes of Ranvier, separated from each other by myelin-covered internodes. On either side of the nodes, axoglial interactions determine the formation of the paranodes and juxtaparanodes. 4.1B is enriched in these two domains, in both central and peripheral myelinated axons [14], [15]. 4.1B immunoreactivity has also been detected in the internodes [14], [16]–[18]. At paranodes and juxtaparanodes, 4.1B associates with two proteins of the NCP (neurexinIV-caspr-paranodin) family, Caspr/paranodin, and Caspr2, respectively [14], [15], [19]. These two proteins are highly homologous and share a juxtamembrane cytoplasmic region which contains a 4.1-binding motif (Glycophorin C, Neurexin IV, Paranodin, (GNP)) [15], [20]. They both bind 4.1B FERM (four point one-ezrin-radixin-moesin) domain *in vitro* and *in vivo* [15]. The localization of 4.1B in peripheral myelinated axons occurs independently of the presence of Caspr/paranodin at paranodes or Caspr2 at juxtaparanodes [17], [19], [21]–[23], suggesting that it might be a primary component of these regions. In the absence of 4.1B the juxtaparanodal localization of Caspr2 is impaired [17], [18]. To further address the role of 4.1B *in vivo* we generated 4.1B knockout mice and studied their phenotype. Our results support a role of 4.1B not only at juxtaparanodes, but also at paranodes and in the internodal region, suggesting that this protein contributes to the maintenance of axon caliber and the myelinated fiber integrity.

## Results

### Protein 4.1B KO mice are viable and do not display major developmental defect

**Figure 1:**
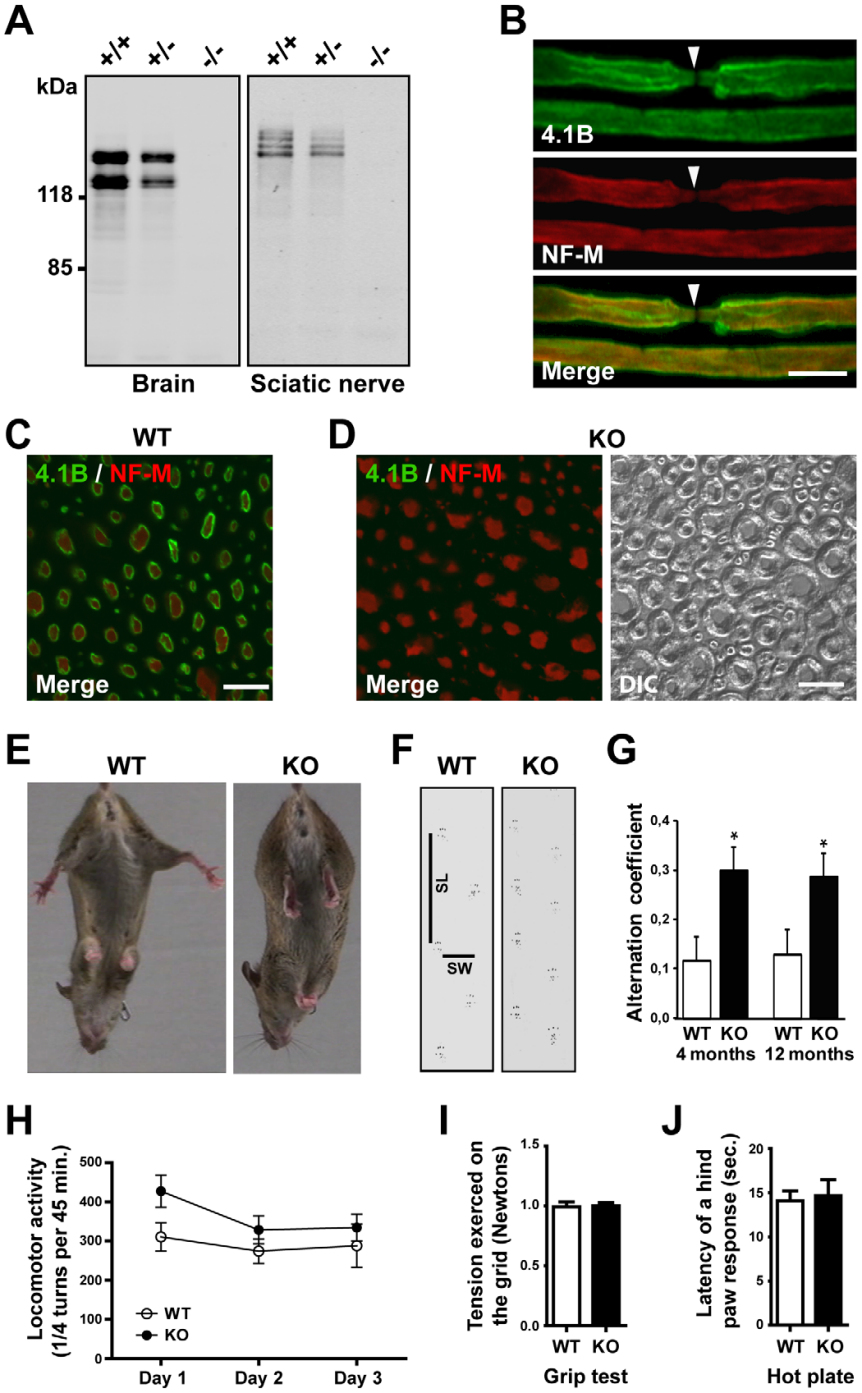
Identification and behavioral phenotype of 4.1B KO mice. **A.** Protein 4.1B expression in brains and sciatic nerves of WT (+/+), 4.1B heterozygous (+/-) and homozygous (-/-) KO mice. **B.** Teased sciatic fibers from an adult WT mouse doubly stained for protein 4.1B (green) and neurofilament subunit NF-M (red). Superposition of the two labels is shown (Merge). 4.1B is localized all along the axolemma of myelinated axons with the exception of the node (arrowhead). **C, D.** Transversal sections of dorsal spinal roots from adult WT (C) and KO (D) mice, doubly stained for protein 4.1B (green) and neurofilament subunit NF-M (red). Superposition of the two labels (Merge, C, D) and differential interference contrast (DIC, D) are shown. 4.1B staining, encircling the NF-M positive axoplasm, is visualized only on the surface of the fibers in the WT mouse (C) and is absent in the 4.1B KO mouse (D). **E.** Limb clasping of a KO mouse compared to a WT littermate. **F.** Representative footprint patterns of 4-month-old WT and KO mice. Vertical and horizontal bars on the WT pattern represent stride length (SL) and stride width (SW) measures, respectively. **G.** Alternation coefficient of 4-month-old WT (*n = *3) and KO (*n = *4) mice, and 12-month-old WT (*n = *4) and KO (*n = *5) mice. **H.** Spontaneous locomotor activities of 4- to 6-month-old WT (*n = *10) and KO (*n = *18) mice measured during 3 consecutive days. **I.** Forelimb grip strength analysis of 4- to 6-month-old WT (*n = *15) and KO (*n = *12) mice. **J.** Heat sensitivity of 4- to 5-month-old WT (*n = *7) and KO (*n = *7) mice. Statistical analysis, 2-way ANOVA followed by Bonferonni post-test (H, *p<0.05), 2-way RM ANOVA (I, *p*>0.05), unpaired *t*-test (J, *P = *0.87). Scale bars: 10 µm.

Mutant mice were obtained using a three-lox recombination strategy [24] to generate a *protein 4.1B^KO3^* allele deleted from the exon 3 of the *Dal-1* gene (Figure S1). Exon 3 of the *Dal-1* gene codes for the first amino acids of the FERM domain of protein 4.1B (Figure S2A). Its deletion was predicted to introduce a premature stop codon and to lead to the production of a small protein unable to interact with NCP-family proteins and the cytoskeleton, if stable. Crossing heterozygous mice generated litters in which the homozygous pups were obtained at a Mendelian ratio (wild type: 26.6%; heterozygous: 46.8%; homozygous: 26.6%; *n = *474) demonstrating that protein 4.1B is not necessary for mouse development. When wild type brain or sciatic nerve were analyzed by immunoblotting, several immunoreactive protein 4.1B bands were observed (Fig. 1A), in agreement with the various reported splice isoforms [3], [4], [18], [25]. These bands were *bona fide* 4.1B since the immunoreactivity completely disappeared in homozygous mutant mice, while intermediate levels were observed in heterozygous mice (Fig. 1A). Interestingly, the apparent molecular weight of these bands was different in brain and sciatic nerves, reflecting a general difference in protein 4.1B isoforms expression between the central (CNS) and the peripheral (PNS) nervous systems as demonstrated by immunoblotting of various peripheral nerves and CNS tissues from mouse (data not shown) and rat (Figure S2B, C).

### Protein 4.1B is localized all along the axolemma of myelinated axons with the exception of nodes of Ranvier

Protein 4.1B was first reported to be enriched in the paranodal and juxtaparanodal regions of PNS and CNS myelinated fibers [14], [15], [21]. 4.1B immunoreactivity was also detected in the internodes of myelinated axons [14], [16]–[18]. However, this immunoreactivity appeared non-homogenously distributed and often less intense than at paranodes and juxtaparanodes. To further characterize the localization of 4.1B, we performed immunolabeling on teased sciatic fibers with tissue fixation and post-fixation conditions different from those previously used (see Materials and Methods). We observed that 4.1B immunoreactivity extended beyond the juxtaparanodal region, lining continuously the axolemma in the internode, with an intensity comparable to that observed at paranodes and juxtaparanodes (Fig. 1B). In agreement with this observation, in transverse sections of dorsal spinal roots from wild type mice (WT), 4.1B immunoreactivity was observed in all fibers as a ring surrounding neurofilaments immunoreactivity (Fig. 1C). All the 4.1B immunoreactivity disappeared in mutant mice (KO) (Fig. 1D). These results demonstrated that protein 4.1B is localized at the vicinity of the axolemma in myelinated fibers, not only in paranodal and juxtaparanodal regions but also all along the entire length of the axon, with the exception of the nodes themselves.

### 4.1B knockout mice display mild ataxia

4.1B mutant mice were normal in size and no significant lethality was observed during the first 6 months of life, whereas in older mutant mice the mortality increased significantly (Figure S3). We examined the behavior of the adult mutant mice. During tail suspension the animals had a posture of hind limb and forelimb clasping (Fig. 1E) reported to occur in mouse models in which there is neurological dysfunction [26], [27]. They also had slight gait abnormalities evidenced by the footprint test (Fig. 1F). The stride length was decreased in young (4-month-old) as well as in older (12-month-old) mice, while the stride width was normal (data not shown). The alternation coefficient, reflecting the uniformity of step alternation, was consequently significantly increased in mutant mice as compared to WT mice (Fig. 1G). By contrast, horizontal locomotor activity was not significantly altered (Fig. 1H). The muscular strength was similar in both genotypes for front limbs (Fig. 1I). No difference in reaction time was observed when mice of two genotypes were placed on a hot plate (Fig. 1J), indicating a lack of major alteration in nociception. Thus, changes in gait without major motor deficit revealed an isolated ataxia in 4.1B knockout mice.

### The localization and levels of paranodal proteins are altered in sciatic nerves of 4.1B knockout mice

**Figure 2:**
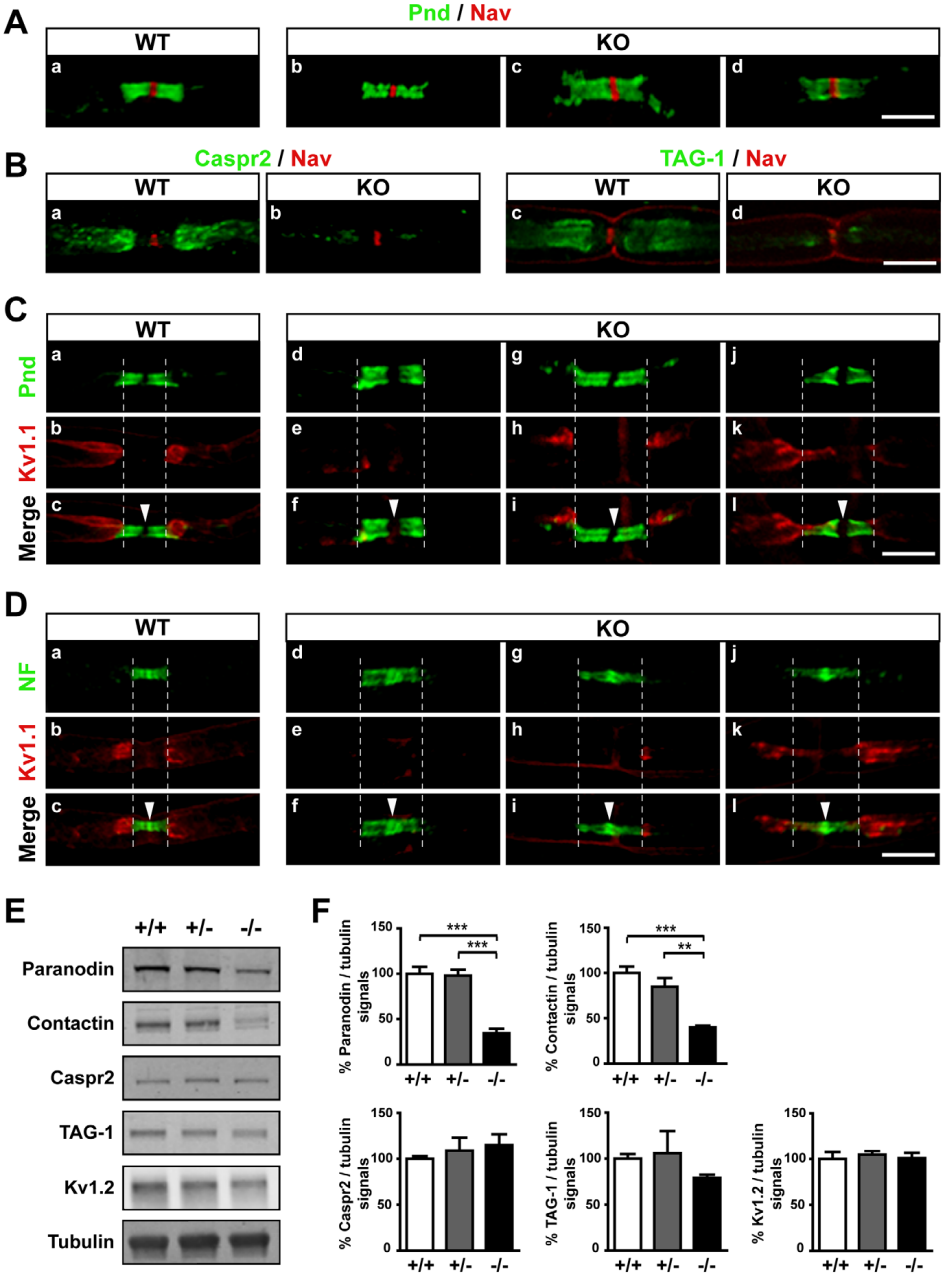
Distribution and expression levels of paranodal and juxtaparanodal proteins in sciatic nerves of 4.1B KO mice. **A**–**D.** Immunolocalization of Na_v_ (red, A, B), Caspr/paranodin (Pnd, green, A, C), Caspr2 (green, B), TAG-1 (green, B), Kv1.1 subunits (Kv1.1, red, C, D), and neurofascin (NF, green, D) in teased sciatic fibers from adult WT and KO mice. Single labels and superpositions (Merge) are shown. Arrowheads, nodes. In KO mice, paranodin and neurofascin labeling appears less homogeneous and not as clearly limited as in WT paranodes (A, C, D). The distribution of Caspr2 and TAG-1 immunoreactivities are markedly decreased at juxtaparanodes (B). In most cases, Kv1.1 are not or weakly enriched in the juxtaparanodal regions (C (d–i), D (d–i)). In other cases, Kv1.1 immunoreactivity at juxtaparanodes seems less severely affected (C (j–l), D (j–l)), but can also be found at paranodes (C (j–l)). **E, F.** Proteins expression in sciatic nerves from adult WT (+/+), heterozygous (+/-) and homozygous (-/-) mutant mice evaluated by immunoblotting (E), and quantified and normalized to β-tubulin signal (F, three mice for each genotype). The levels of paranodin and contactin are significantly decreased in homozygous mutant mice compared to WT mice, whereas the levels of the three juxtaparanodal proteins are not significantly altered. Statistical analysis, 1-way ANOVA followed by Bonferroni's multiple comparison test, **p<0.01, ***p<0.001. Scale bars: 10 µm.

Since protein 4.1B is associated with Caspr/paranodin at paranodes [15], we examined the distribution in teased fibers of sciatic nerves of several proteins which are enriched in nodal and paranodal regions. In 4.1B knockout mice, the distribution of sodium channels (pan-Nav antibody) was normal (Fig. 2A, B), as well as that of other nodal markers including ankyrin G (data not shown). In contrast, paranodin labeling appeared less homogeneous and not as clearly limited as in WT paranodes (Fig. 2A, C). In some nodes, paranodin immunoreactivity extended towards the internodes, an aspect not observed in WT mice. Similar observations were done using antibodies against contactin (data not shown), which is associated in cis with paranodin in the axonal paranodal regions [28]. The labeling for the 155-kDa isoform of neurofascin, the partner of the paranodin/contactin complex localized in the glial membrane [29]–[31], was similarly altered (Fig. 2D, NF). Immunoblotting showed that the levels of paranodin and contactin were significantly decreased in the sciatic nerves of homozygous protein 4.1B mutant mice, but were unaltered in heterozygotes (Fig. 2E, F), indicating that intermediate levels of protein 4.1B were sufficient to maintain normal levels of contactin and paranodin. Since the intensity of the immunoreactivity of paranodin and contactin at paranodes did not appear significantly diminished, these results also suggest that a larger proportion of the total pool of these proteins was targeted to the paranodal axolemma in the absence of 4.1B. Similar alterations of paranodin distribution were observed in KO mice during development from post-natal day 5 to adulthood (data not shown), indicating that the mislocalization of the paranodal proteins occurred during development.

### The juxtaparanodal proteins are mislocalized but their levels are unchanged in sciatic nerves of 4.1B knockout mice

We then examined the organization of axonal proteins at juxtaparanodes, where Caspr2 is associated with the glycosylphosphatidyl- (GPI-) anchored protein TAG-1 in cis, in the axolemma, and where this complex interacts in trans with another molecule of TAG-1 in the Schwann cell membrane [22], [32]. These multimolecular complexes are important for the targeting of *Shaker*-type K^+^ channels (Kv1.1 and Kv1.2 subunits) [22], [32]. In 4.1B mutant mice the distribution of Caspr2 and TAG-1 immunoreactivities were markedly decreased at juxtaparanodes, with scattered clusters of weak labeling (Fig. 2B). The distribution of Kv1.1 was also altered, but more variable (Fig. 2C, D). In most cases, Kv1.1 was not or weakly enriched in the juxtaparanodal regions (Fig 2C (d–i), D (d–i)). In other cases, Kv1.1 immunoreactivity at juxtaparanodes seemed less severely affected (Fig. 2C (j–l), D (j–l)), but could also be found at paranodes (Fig. 2C (j–l), D (j–l)). Immunoblotting showed that, in contrast to what was observed for paranodal proteins, the levels of the three juxtaparanodal proteins were not significantly altered in sciatic nerves from mutant mice (Fig. 2E, F). Similar alterations of Caspr2 and TAG-1 distribution were observed at post-natal day 8 in KO pups (data not shown).

### Ultrastructural alterations of paranodal and juxtaparanodal regions in the absence of protein 4.1B

**Figure 3:**
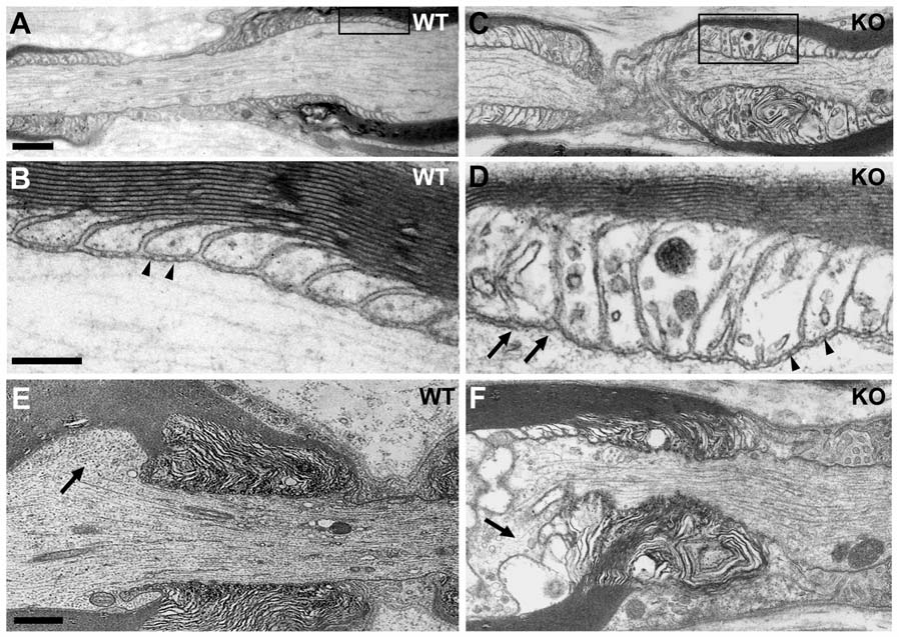
Ultrastructure of nodal regions in 4.1B KO mice. Electron micrographs of longitudinal sections of sciatic nerves, at the level of nodes of Ranvier, from WT (A, B (higher magnification of box in A), E) and KO mice (C, D (higher magnification of box in C), F). In the WT mouse, paranodal loops are symmetrical across the axon (A), and transverse bands are visible at the paranodal junctions (B, arrowheads). In the paranodal regions of the KO mouse irregular paranodal loops are frequently observed (C, D), transverse bands between the axolemma and some glial loops are not apparent in some places (D, arrows), but clearly visible in others (D, arrowheads). At juxtaparanodes, cytoplasmic Schwann cell protrusions in the axon are observed in mutant mice (F, arrows) but not in WT mice (E, arrows). Scale bar: A, C, 0.7 µm; B, D, 0.3 µm; E, F, 0.9 µm.

At paranodes, the myelinating glial cell lateral loops spirally wrap around the axon, and the glial membrane is tightly attached to the axolemma by septate-like junctions. Five to ten nodes of Ranvier were examined in longitudinal sections of sciatic nerves from 4- and 13-month-old mice of each genotype (2 mice per group) at the ultrastructural level. In WT mice, the paranodal myelin loops were tightly and regularly positioned at paranodes, with a symmetrical organization and size across the heminode (Fig. 3A, B). Transverse bands were clearly visible (Fig. 3B, arrowheads). In KO mice, paranodal loops were frequently irregular but well organized transverse bands were still visible in some regions of the paranodes (Fig. 3D, arrowheads), demonstrating that 4.1B is not necessary for the formation of septate-like junctions. However, abnormal interactions between axons and glial cells were observed in KO mice as compared to WT mice (Fig. 3E and F, arrows). At juxtaparanodes, cytoplasmic Schwann cell protruded in the axon (Fig. 3F, arrows; 4-month-old mice, 19 juxtaparanodal regions affected out 32 observed; 13-month-old mice, 16 juxtaparanodal regions affected out 30 observed).

### The distribution of axonal βII spectrin is altered in the absence of protein 4.1B

**Figure 4:**
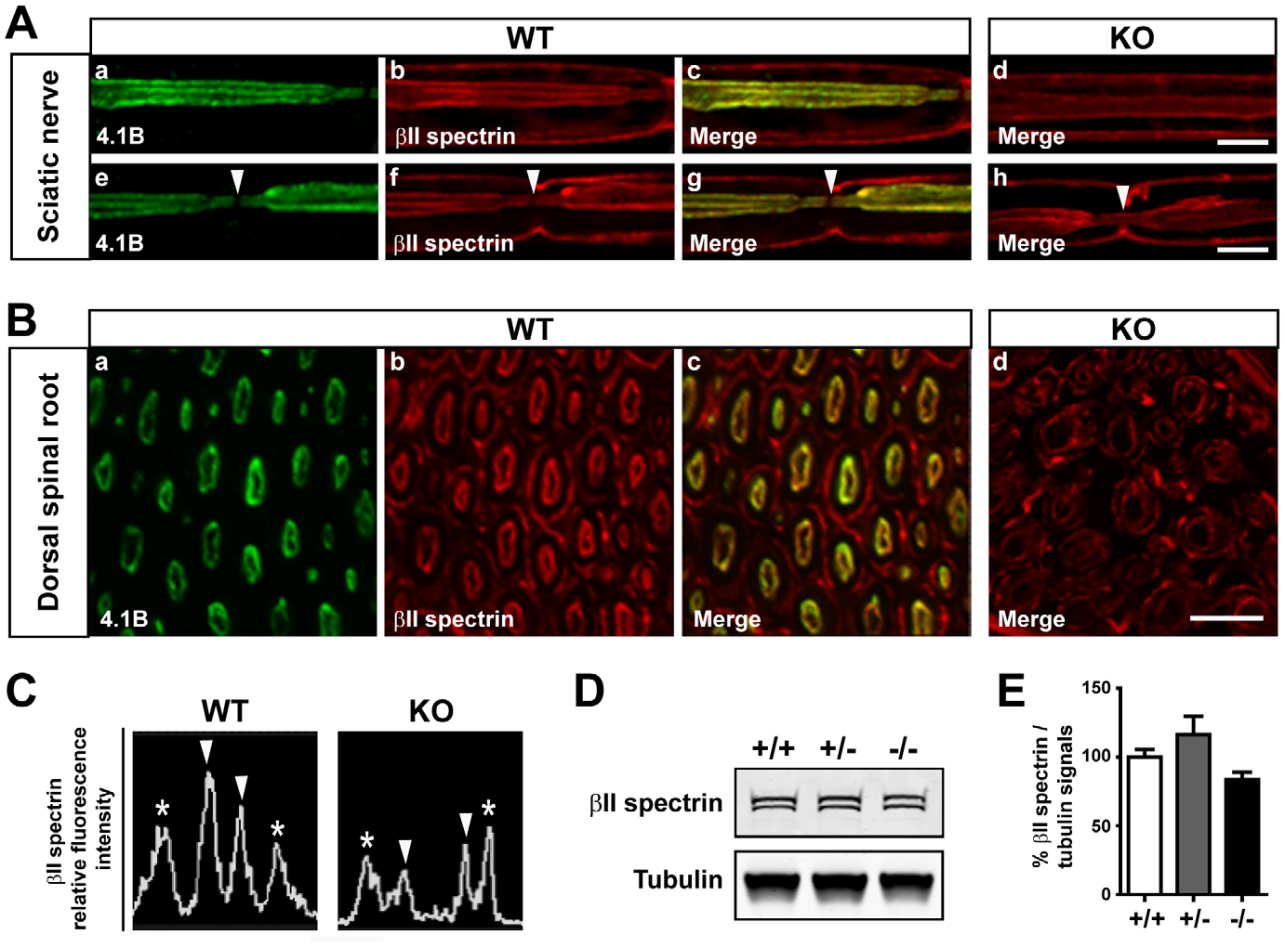
Abnormal distribution of βII spectrin in axons of 4.1B KO mice. **A.** Teased sciatic fibers from adult WT (a–c and e–g) and KO (d, h) mice double stained for protein 4.1B (green) and βII spectrin (red). Superpositions of the two labels (Merge, WT, c, g, KO, d, h) are shown. As for protein 4.1B, in WT mice βII spectrin immunoreactivity lined the axolemma along the whole length of the axon with the exception of the node (e–g, arrowheads). In mutant mice some βII spectrin immunoreactivity is still detectable along the axolemma (d, h), and at the node (h, arrowhead). **B.** Transversal sections of dorsal spinal roots from adult WT (a–c) and KO (d) mice doubly stained for protein 4.1B (green) and βII spectrin (red). Superpositions of the two labels (Merge, WT, c, KO, d) are shown. Concentric circles of axonal and glial βII spectrin immunoreactivity are observed in WT mice, with the inner (axonal) one being also labeled with 4.1B antibodies (a–c). In mutant mice, the inner ring of immunoreactivity is much less regular and intense relatively to the glial ring of immunoreavtivity (d). **C.** Representative fluorescence intensity profiles of βII spectrin labeling of dorsal spinal root myelinated fibers from WT and KO mice. Arrowheads, axonal labeling; asterisks, glial cells abaxonal membrane labeling. **D, E.** βII spectrin expression in sciatic nerves from adult WT mice (+/+), heterozygous (+/-) and homozygous (-/-) mutant mice evaluated by immunoblotting (D), and quantified and normalized to β-tubulin signal (E, three mice for each genotype). Statistical analysis, 1-way ANOVA followed by Bonferroni's Multiple Comparison Test. No significant change was observed between the different genotypes. Scale bars: 10 µm.

A major function of proteins 4.1 is to anchor the cortical spectrin-based cytoskeleton to the cell membrane and, thus, contribute to the control of cell shape [1]. βII spectrin immunoreactivity was previously reported at paranodes and juxtaparanodes in optic and sciatic nerves, as well as all along the optic axons [16]. An association between protein 4.1B and βII spectrin was also detected in mouse brain lysates [16]. Thus, we carefully examined the distribution of βII spectrin in WT and mutant mice (Fig. 4). When we fixed WT dissociated sciatic fibers using the similar conditions used for 4.1B detection, we noticed that, as for protein 4.1B, βII spectrin immunoreactivity lined the axolemma along the whole length of the axon (Fig. 4A (a–c)), with the exception of the node of Ranvier (Fig. 4A (e–g, arrowheads)). Immunoreactivity was also observed along the abaxonal membrane of Schwann cells (Fig. 4A (b, f)). In mutant mice some βII spectrin immunoreactivity was still detectable along the axolemma (Fig. 4A (d, h)), but was also detectable in the node (Fig. 4A (h, arrowhead)). To better visualize βII spectrin localization we examined transversal sections of dorsal spinal roots (Fig. 4B (a–c)). In these preparations, concentric circles of axonal and glial βII spectrin immunoreactivity were observed in WT mice, with the inner (axonal) one being also labeled with 4.1B antibodies. In mutant mice, the inner ring of immunoreactivity was much less regular and intense as compared to the glial ring of immunoreactivity (Fig. 4B (d), 4C). However, the levels of βII spectrin were not significantly decreased in 4.1B mutant mice compared to WT mice (Fig. 4D, E). These results showed that βII spectrin is also localized all along the axon and that its distribution is altered in the absence of protein 4.1B, suggesting that 4.1B mutant mice could present axonal internodal defects.

### 4.1B KO mice present internodal axon and myelin defects

**Figure 5:**
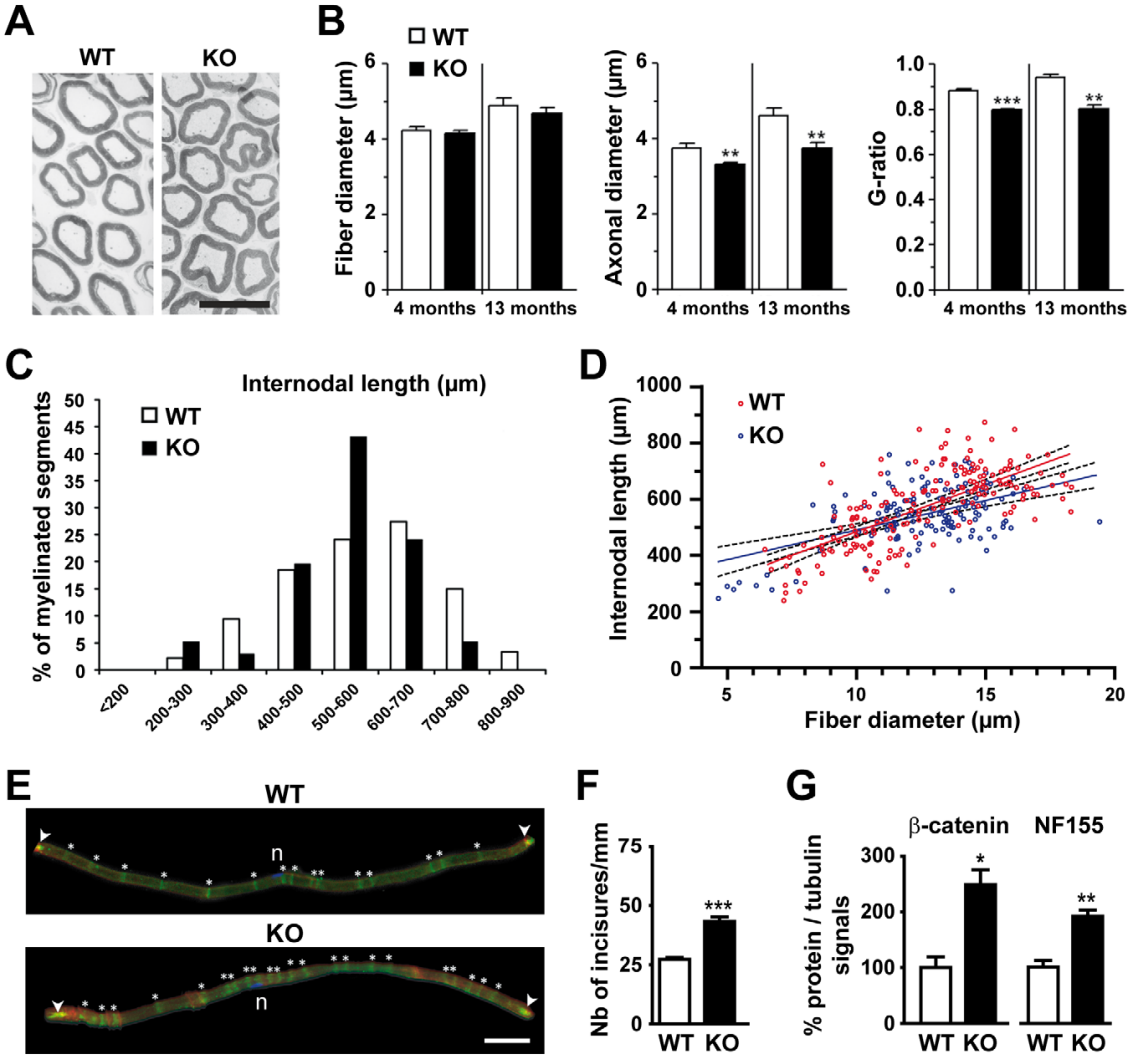
Internodal abnormalities in phrenic and sciatic nerves from 4.1B mutant mice. **A.** Electron micrographs from transverse sections of phrenic nerves of 4-month-old WT and KO mice. **B.** Fiber diameter, axonal diameter, and G-ratio in phrenic nerves of 4- or 13-month-old WT and KO mice. The size of the axons was slightly reduced and the thickness of the myelin sheath increased in KO mice. Statistical analysis, Mann-Whitney test, ** *p*<0.01, ***p<0.001 (B). **C.** Internodal length class histogram distribution of myelinated segments from sciatic nerves of 6-month-old WT and KO mice. Internodal lengths are less spread in KO than in WT mice, with more than 40% of the fibers with a fiber diameter of 500–600 µm. Statistical analysis, Chi 2 = 27.77, *p*  = 0.0001. **D.** Correlation of internodal length with nerve fiber diameter. The slopes are significantly different between the two genotypes: WT, 33.5 ± 2.5 (*r^2^ = *0.49); KO, 21.0 ± 3.2 (*r^2^ = *0.24). Linear regression, *p*<0.01. **E.** Visualization of Schmidt-Lanterman incisures (SLIs, NF155 labeling, in green, asterisks) and nodes (Na_v_ labeling, in red, arrowheads) in teased sciatic fibers from adult WT and KO mice. n, nuclei of the Schwann cells stained with DAPI (blue). **F.** Quantification of the average number of SLIs per mm in sciatic nerves from adult WT and KO mice. Statistical analysis, two-tailed *t*-test, *** *p*<0.0001. **G.** NF155 and β-catenin expression in sciatic nerves from adult WT and KO mice evaluated by immunoblotting (data not shown), and quantified and normalized to β-tubulin signal (three mice for each genotype). Levels of β-catenin and NF155 are significantly increased in KO mice compared to WT mice. Unpaired *t*-test, * *p*<0.05, ** *p*<0.005. Scale bars: A, 10 µm; E, 50 µm.

We started to study the internodal region in transverse sections of phrenic nerves. In semi-thin sections at low magnification (Fig. 5A), the number of fibers was not decreased in 4-, 8- and 13-month-old knockout mice, as compared to WT littermates (data not shown). Analysis of ultrathin sections at low magnification showed no change in the fiber diameter at 4 and 13 months (Fig. 5B), whereas the axonal diameter was decreased in knockout mice at both ages (−11.7% and −19.7%, respectively, Fig. 5B). Consequently, the myelin thickness was relatively increased in the mutant mice, as indicated by a slight decrease in the G-ratio (Fig. 5B).

To investigate whether KO mice present additional internodal alterations, we examined dissociated fibers from the sciatic nerve, which display a wide range of caliber. We analyzed the internodal length determined as the distance between two nodes stained for Na_v_ and found a slight decrease in the mean value of this parameter (WT, 570 ± 10 µm, KO, 542 ± 8.9 µm; unpaired *t*-test, *p<0.05; WT, *n = *179, KO, *n = *137). A more detailed analysis revealed that the distribution histogram of internodal lengths was less spread in KO than in WT mice, with more than 40% of the fibers with an intermodal length of 500–600 µm (Fig. 5C). When we plotted the internodal length as a function of the fiber diameter, we observed a significant correlation (Fig. 5D), as expected [33]. However, the slopes were slightly but significantly different between the two genotypes, with a lesser increase of the internodal length as a function of fiber diameter in KO mice.

We also quantified the number of Schmidt-Lanterman incisures (SLIs), which are helicoidal cytoplasmic channels through the compact myelin of Schwann cells. SLIs are characterized by the presence of Schwann cell autotypic contacts where are localized cadherins, catenins, claudins, connexins and neurofascin 155-kDa isoform (NF155) [34], [35]. We visualized the SLIs in teased sciatic fibers by NF155 immunostaining. We observed that the number of SLIs/mm was significantly increased in 4.1B mutant mice (Fig. 5E, F). In agreement with this finding, immunoblot analysis revealed increased levels of β-catenin and NF155 (Fig. 5G).

Internodal changes were not accompanied by dramatic changes in the major protein components of myelin or axonal cytoskeleton. No alteration in the levels of two major proteins of the myelin sheath, P0 and myelin basic protein (MBP), the three subunits of neurofilaments, NF-L, NF-M, and NF-H, as well as phosphorylated NF-H (NF-H-Ph) and actin, was observed in sciatic nerves of WT, heterozygous, and homozygous mutant mice (Figure S4A-D).

### Motor axons are altered in the absence of protein 4.1B

**Figure 6:**
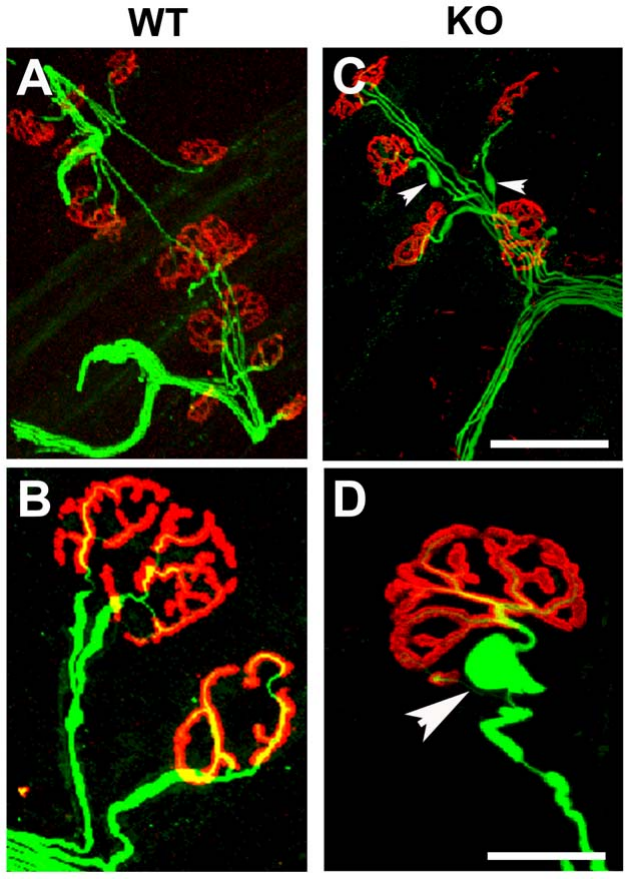
Alteration of preterminal axons of motor endplates in 4.1B KO mice. *In toto* immunostaining of neuromuscular junctions on preparations of teased fibers from WT (A, B) and KO (C, D) mice with rhodamine-conjugated α-bungarotoxin (red) and antibodies directed against neurofilament subunit NF-M (green). NF-M labeling revealed irregular caliber with dilated segments in myelinated preterminal axons of the KO mouse (C, D, arrowheads). Scale bars: A, C, 75 µm; B, D, 15 µm.

To further investigate how the absence of protein 4.1B could affect the morphology of axons, we studied the motor axons in skeletal muscle where they reach their target. Labeling of neuromuscular junctions (NMJ) was performed on hind limb muscles (*tibialis anterior* or *extensor digitorum longus*) from WT and 4.1B mutant mice. Whole mount preparations of muscle fibers were stained with rhodamine-conjugated α-bungarotoxin and a monoclonal antibody directed against the neurofilament subunit NF-M to label the acetylcholine receptors (AChR) and axons, respectively (Fig. 6). The WT preterminal axons had a relatively regular caliber (Fig. 6A, B). In contrast, an irregular swollen aspect of NF-M-positive preterminal axons was observed in mutant mice, alternating with narrow segments (Fig. 6C, D, arrowheads). These axonal swellings were only observed in preterminal myelinated axons and not in terminal axons in the synaptic gutter stained with α-bungarotoxin. These findings further indicated an abnormal axonal structure in the absence of protein 4.1B.

### 4.1B knockout mice nerves have a normal conduction but display transient temperature sensitive hyperexcitability

We finally investigated whether the morphological abnormalities observed in mutant mice may influence peripheral nerve functions. To this end, we first examined the electrophysiological properties of the sciatic nerves of 5-month-old mutant mice compared to WT littermates. Recording from sciatic nerves did not reveal any alteration in the shape of compound action potentials (CAPs) at 35°C (Figure S5A) or 22°C (data not shown). Although the conduction velocity (CV) of CAPs was slightly decreased in mutant mice, the difference with their WT littermates was not significant (Table 1). The CV of C fibers was also not significantly affected and no conduction block was observed (data not shown). The refractory period was virtually identical in both genotypes at 35°C (Figure S5B) or 22°C (data not shown). The recruitment of the CAPs was similar in WT and mutant mice (Figure S5C). These basic parameters were also unaltered in the presence of increased extracellular K^+^ (9 mM) or of K^+^ channel blockers tetraethylammonium (TEA, 10 mM) and 4-aminopyridine (4-AP, 500 µM), alone or in combination, or following repeated stimulation (data not shown).

**Table 1:** Electrophysiological characteristics of sciatic nerves of WT and KO mice at 35°C and 22°C.

T°C	Genotype	CAP^1^ amplitude (mV)	CV_Vmax_ 2 (m.s^−1^)	CV_V1/2_ (m.s^−1^)	Duration_V1/2_ (ms)	CAP area (10^−6^V.s)
35°C	WT	11.6±3.3	39.4±6.8	66.6±9.0	0.37±0.06	2568±626
35°C	KO	10.0±4.8	34.4±6.7	57.3±11.3	0.37±0.04	2264±978
22°C	WT	10.8±3.3	19.6±3.0	39.8±4.9	0.91±0.07	6024±2165
22°C	KO	10.6±3.2	18.1±1.7	36.0±4.6	0.89±0.05	5316±1726

The data were recorded from 9 and 10 sciatic nerves from 5-month old WT and KO mice, respectively. Electrophysiological characteristics of sciatic nerves of WT and KO mice are not significantly different (two-tailed *t*-test for two samples of equal variance).

1 CAP, compound action potential.

2 CV, conduction velocity.

**Figure 7:**
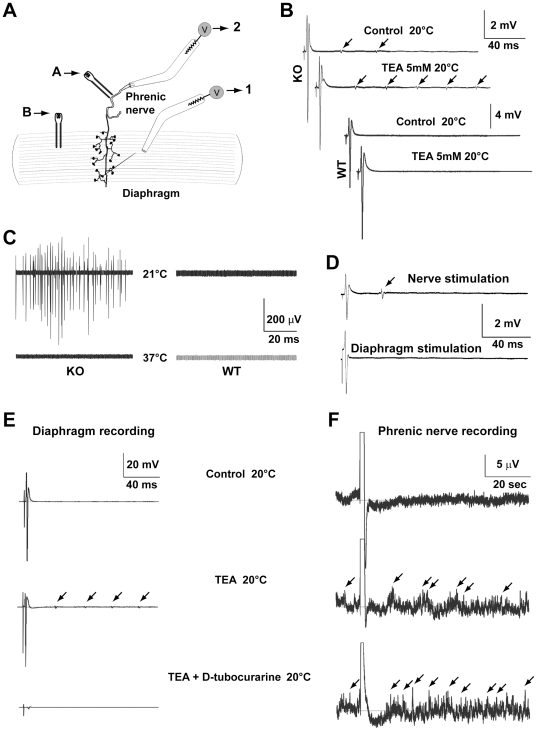
Neuromuscular electrophysiological properties of WT and 4.1B KO mice. **A.** Nerve (CAP) and muscle (CMAPs) compound action potentials recording protocol. The CMAPs were recorded with a surface electrode pressed on the diaphragm (1) after stimulation of the phrenic nerve (A) or the diaphragm (B) using bipolar electrodes. The phrenic nerve CAPs (2) were simultaneously recorded from the cut end with a suction electrode. **B.** Typical CMAPs recorded from diaphragms of P21 WT and KO mice after phrenic nerve stimulation at 20°C in the absence and in the presence of the potassium channel blocker TEA. **C.** Muscle spontaneous activity recorded from P21 WT and KO mice at 21°C and 37°C in the presence of the potassium channel blocker TEA. **D.** Repetitive activity is recorded in the diaphragm of KO mice following stimulation of the phrenic nerve (upper trace, arrow) but not of the muscle fibers (lower trace). **E.** In the diaphragm, repetitive activity is elicited by the blockade of potassium channels with TEA (5 mM, arrows), and prevented in the presence of D-tubocurarine (20 µM), indicating that it originates in the axons. **F.** TEA also elicits repetitive activity (arrows) in the phrenic nerve axons insensitive to D-tubocurarine.

We next examined whether 4.1B mutant animals exhibited neuromuscular alterations, and we recorded the diaphragm compound muscle action potential (CMAPs) in response to the stimulation of the phrenic nerve in 3-week-old mice (Fig. 7A). CMAPs had a typical triphasic shape in both mutant and WT littermates (Fig. 7B). Following a single stimulation, repetitive activity was recorded in 4.1B KO mice at 20°C, but neither in WT mice (Fig. 7B) nor at 37°C (not shown). In addition, spontaneous muscle activity was observed in mutant mice at 20°C, but neither at 37°C nor in WT animals (Fig. 7C). These data indicated that 4.1B-deficient animals presented cooling-induced hyperactivity similar to that observed in mice deficient for the voltage-sensitive potassium channels Kv1.1 (*Kcna1*-null mice) [36], [37]. Repetitive muscle contractions were not observed after direct stimulation of the diaphragm muscle, indicating they did not originate in the muscle (Fig. 7D). Similarly to *Kcna1*-null mice, we found that the repetitive and spontaneous activities were markedly increased in the presence of 5 mM TEA at 20°C (Fig. 7B, E). In particular, TEA elicited repetitive activities in phrenic nerve axons (Fig. 7F), suggesting that hyperactivity had an axonal origin and back-propagated in the axons. This hypothesis was further confirmed by the blockade of muscular repetitive activity induced by phrenic stimulation in the presence of D-tubocurarine (Fig. 7E), which blocks nicotinic acetylcholine receptors and neuromuscular transmission, whereas this treatment had no effect on the repetitive activity recorded in the nerve (Fig. 7F). When we recorded in mice at various ages from 2 to 25 weeks, we observed the evoked and spontaneous hyperactivity only around 3 weeks (Figure S6), showing that this was a transient phenomenon during development. These results demonstrate that in the absence of protein 4.1B, a transient nervous hyperexcitability arises that is masked almost completely by compensatory effects of K^+^ channels, as revealed by TEA blockade.

## Discussion

The results reported here demonstrate the importance of protein 4.1B in the organization of myelinated axons. It had been previously shown that 4.1B interacts with two proteins enriched in paranodal and juxtaparanodal regions, Caspr/paranodin and Caspr2, respectively [15] through their GNP motif [20]. The present study supports a functional role of these interactions and expands the role of 4.1B to the internodal regions.

The paranodes serve as a membrane barrier that excludes juxtaparanodal components from the node [19], [23], [30], [38], [39]. In 4.1B mutant sciatic nerves we observed that the absence of 4.1B did not prevent the targeting of paranodin or its axonal partner contactin at paranodes. However, the levels of expression of these proteins were decreased and their distribution was altered. Paranodes appeared non homogeneous and with less clearly defined limits than in WT mice. This altered distribution of the paranodal proteins affected the localization of the juxtaparanodal K^+^ channels which were at times mislocalized at the paranodes. Thus, our results support a role of protein 4.1B in the membrane trafficking and stabilization of the paranodal complexes in the axonal membrane, contributing to the paranodal membrane barrier establishment. It is likely that protein 4.1B could play this role through its association with the spectrins (αII and βII spectrins) localized in the paranodes [16]. Interestingly, at the ultrastructural level, paranodal transverse bands between the axolemma and glial loops were still detectable in some places in 4.1B mutant mice, demonstrating that 4.1B is not necessary for their formation.

Our observations on the role of 4.1B on paranodes organization in the PNS are in agreement with previous results showing that stable expression and localization of paranodin at the paranodes in transgenic mice requires its intracellular 4.1-binding motif [21]. Furthermore, they are in accordance with the results observed recently by others in mutant mice obtained using a two-lox recombination strategy to generate a mutant allele deleted from the exon 6 of the *4.1B* locus (*β-Act-Cre;4.1B^Flox^* mice) [18]. The distribution of paranodin and NF155 was also disorganized in the paranodal regions of these mice. Paranodes often displayed severe morphological defects, but array of transverse bands could be found. However, our observations differ from those reported in an alternative model of mice deficient for the expression of 4.1B generated using a conventional recombination method to delete the exon 3 of the *DAL-1* gene [40]. In these mice (referred to below as “4.1B-null” mice) paranodin, contactin, and NF155 were found to be correctly localized in the paranodal regions and Kv1 channels were not detected at the nodes or the paranodes [17], [18]. A possible explanation for the differences between mutant mouse models could be related to the differences in their *4.1B* locus mutations and/or their genetic backgrounds. Supporting this hypothesis, “4.1B-null” mice did not show neurological abnormalities and no difference in the longevity compared to WT littermates [40], in contrast with our 4.1B KO mice. It is also possible that other types of protein 4.1 compensate for the absence of 4.1B. Although we did not detect an increase in 4.1N in sciatic nerve extracts (Goutebroze, unpublished observations), the normal levels of this isoform, or possibly others may be sufficient to take over some of the functions of 4.1B.

In the absence of protein 4.1B, we also observed that the distribution of the main proteins of juxtaparanodes was profoundly altered. The enrichment of both Caspr2 and TAG-1 was almost completely lost, showing that protein 4.1B is required for the correct localization of these molecules, in agreement with the results observed by other groups using different mouse models [17], [18], [21]. In addition, we observed that Caspr2 and TAG-1 were not detected at juxtaparanodes during development, suggesting that protein 4.1B controls the accumulation of Caspr2 at the juxtaparanodes rather than its stabilization in the axonal membrane. Consistent with this hypothesis, the levels of Caspr2 and TAG-1 were not decreased in adult mutant mice, showing that there was no destabilization, at least no degradation of the Caspr2/TAG-1 complexes, and that they were probably redistributed along the internodes. Importantly, in TAG-1 mutant mice the distribution of 4.1B was not altered, showing that its enrichment is not dependent on the Caspr2/TAG-1 membrane proteins complexes [22]. Thus, our results strengthen the hypothesis that 4.1B plays the primary role in the localization of these complexes to juxtaparanodes. Moreover, our observations showing Schwann cells protrusions in the axon in nearly half of sciatic nerve juxtaparanodal regions observed in the mutant mice, indicate that 4.1B might be required, directly or indirectly, in the structural organization of the juxtaparanodes.

Important components of juxtaparanodal membranes are the *Shaker*-type K^+^ channels Kv1.1 and Kv1.2 [41]. Kv1.1/2 channels localization depends in part on both TAG-1 and Caspr2 [22], [32]. Caspr2 was originally thought to control Kv1.1/2 clustering through their PDZ binding motif and through the PDZ proteins, PSD93 and PSD95, expressed at juxtaparanodes. However, Kv1.1/2 clustering is not affected in mice deficient for PSD-93 and/or PSD-95 [42], [43] and in mutant mice expressing Caspr2 lacking its carboxy-terminal PDZ-binding motif [42]. Instead, Kv1.1/2 clustering appears to require the 4.1-binding region of Caspr2 [17]. We found that the enrichment of Kv1.1/2 at juxtaparanodes was highly altered in 4.1B knockout mice, with the exception of some remaining clusters. Thus, our results also support the important role of protein 4.1B in Kv1.1/2 channels accumulation at juxtaparanodes.

Interestingly, the electrophysiological phenotype of 4.1B mutant mice is likely to result in part from the mislocalization of these K^+^ channels. Although the conduction velocity, refractory period, and CAP shape were not significantly altered in sciatic nerves, we observed repetitive activities in the diaphragm following stimulation of the phrenic nerve, as well as spontaneous activity. These manifestations of hyperexcitability, corresponding to neuromyotonia/myokymia, were likely due to the repetitive or spontaneous discharge originating within peripheral axons. This hypothesis is supported by the similarities with the phenotype observed in mice lacking Kv1.1 channels, in which abnormalities were ascribed to the lack of channel at the transition zone in muscular nerve terminals [36]. Interestingly a sharp age-dependence of the phenotype was observed in the Kv1.1 mutant mice [36], as in 4.1B mutant mice. This phenomenon may be due to myelin and/or paranodes maturation during development insofar as a role in the prevention of aberrant excitation as myelination proceeds has been attributed to Kv1.1/2 channels [44]. Importantly, hyperexcitability was increased in the presence of a K^+^ channel blocker, TEA, which blocks nodal KCNQ2/KCNQ3 channels [45], [46], suggesting that nodal K^+^ channel activity may compensate for the mislocalization of juxtaparanodal Kv1 channels. Thus, our results indicate that 4.1B is crucial for the precise localization and developmental function of Kv1.1/2 channels, and that 4.1B alterations lead to a channelopathy.

Another important observation in the 4.1B mutant mice was the existence of multiple abnormalities in the internodal region. We confirmed that 4.1B localizes in the internodal axons and further demonstrated that 4.1B immunoreactivity was detected all along the internodal axolemma. In 4.1B KO mice various alterations of the internodal axons were observed including a slight decrease in the axonal diameter in the phrenic nerve and a decreased variety of fiber diameters in dissociated fibers of sciatic nerve (data not shown). These results suggest that 4.1B is required for the precise adjustment of axon caliber. Additional observations suggested that axonal integrity was altered: the terminal axons caliber was irregular at the neuromuscular junction with localized swellings. One of the best characterized functions of proteins of the 4.1 family is to anchor β spectrins [1] and protein 4.1B was shown to interact with βII spectrin [16]. We found that βII spectrin immunoreactivity was detected like 4.1B all along the internodal axolemma in sciatic nerve, whereas this cortical enrichment was altered in the absence of 4.1B. βII spectrin can associate with neurofilaments and may promote the association of intermediate filaments with the plasma membrane and contribute to the organization of neurofilaments [47]–[49]. Since neurofilaments control axonal radial growth with their subunit composition playing a crucial role in determining normal axonal calibers [50], 4.1B might control axonal caliber by controlling intermediate filaments organization through its association with βII spectrin. Interestingly, the internodal length which is normally proportional to axon caliber was also slightly decreased. Moreover, although no protein 4.1B labeling was detected in Schwann cells, several abnormalities were observed at their level including a slight increase in myelin thickness and an increase in the number of SLIs. These modifications suggest that the normal interactions between axons and Schwann cells could be altered, with secondary alterations in myelinating cells. In line of this latter possibility, the protein 4.1B/DAL-1 interacts with the nectin-like proteins (Necl) which encompass a GNP motif [51] and mediate heterophilic axo-glial interactions along the internode [52], [53]. These molecules may provide anchoring sites for 4.1B along the axolemma.

In conclusion, our results show that protein 4.1B is an important component of axons, not only at paranodes and juxtaparanodes, but also in the internodal region. In fact the nodes are the only axonal region completely devoid of 4.1B. The absence of protein 4.1B has different consequences depending on the regions concerned. At paranodes 4.1B is not required for the targeting of the axoglial complexes but for their stabilization in the axonal membrane. By contrast, the presence of 4.1B is necessary for the accumulation of Caspr2, TAG-1, and Kv1.1 at juxtaparanodes. Finally, the abnormalities of internodal regions in 4.1B mutant mice show that, presumably in interaction with βII spectrin, 4.1B is involved in adjustment of axonal diameter. Thus, our study demonstrates that protein 4.1B is an important player in the axonal organization of myelinated fibers.

## Materials and Methods

### Protein 4.1B mouse gene knockout mice

Mutant mice were generated by a conditional mutagenesis strategy described in Figure S1. Briefly, three *loxP* sites flanking sequentially the exon 3 of the *DAL-1* gene and a PGK/*Hygromycin*/*GFP* cassette were introduced by homologous recombination in embryonic stem cells (ES). Recombinant mutant ES cells (hygromycin B-resistant and GFP-positive) were injected into C57BL/6 blastocysts and crossed with FVB/N mice to produce outbred heterozygous offspring *(protein 4.1B^flox3HygGFP/+^* mice). These were crossed with *EIIACre* deletor mice [54] to generate *protein 4.1B^KO3/+^* mice. *Protein 4.1B^KO3/+^* mice were intercrossed to obtain the mice referred as “4.1B KO mice” in the manuscript. All experiments were performed in accordance with the guidelines of the French Agriculture and Forestry Ministry for handling animals (Decree n° 87–848). The laboratory was approved to carry out animal experiments by the Direction Départementale des Services Vétérinaires de Paris, Service de la Protection et de la Santé Animales et de la Protection de l′Environnement (licence B75-05-22). The experimental protocols were approved by the Institut du Fer à Moulin local review board. The principal investigators had a personal agreement (JA Girault, licence 75–877; L Goutebroze, licence 75–1533).

### Antibodies

The anti-4.1B antibody was generated by immunizing rabbits with the amino acids residues P_612_-L_804_ of rat KIAA0987 (Figure S2) fused to GST and purified by affinity. Rabbit polyclonal antibodies (pAb) against paranodin (L51), Caspr2, TAG-1 (TG2), contactin/F3 and NF155, and the mouse monoclonal antibody (mAb) against myelin protein zero (P0), have been described previously [15], [55]–[58]. The other antibodies were from the following sources: voltage-gated Na^+^ channel α subunit mouse mAb (PAN Nav, clone K58/35), β-catenin rabbit pAb, neurofilament 68 mouse mAb (NF-L, clone NR4), neurofilament 160 mouse mAb (NF-M, clone NN18) and neurofilament 200 rabbit pAb (NF-H): Sigma-Aldrich (St Louis, MO); Kv1.1 α subunit mouse mAb (clone K20/78): Antibodies Incorporated (Davis, CA); neuronal class III β-tubulin mouse mAb (TUJ1) and phosphorylated neurofilament 200 mouse mAb (SMI-31): Covance (Berkeley, CA); βII spectrin mouse mAb (clone 42): BD Biosciences (San Jose, CA); myelin basic protein rat mAb (MBP, clone 12): AbD Serotec (Oxford, UK); monoclonal FITC-conjugated sheep anti-rabbit antibodies: Eurobio (Courtaboeuf, France); anti-mouse and anti-rabbit IRDye™800CW-conjugated or IRDye™700CW-conjugated donkey antibodies: Rockland Immunochemicals (Gilbertsville, PA). Cy3-conjugated goat anti-mouse antibodies and rhodamine-conjugated α-bungarotoxin were from Molecular Probes (Leiden, Netherlands).

### Lysates preparation and immunoblotting

Tissues lysates were prepared using a lysis buffer composed of 10 mM NaPi buffer pH 7.8, 59 mM NaCl, 1% Triton-X100, 0.5% deoxycholate, 0.1% SDS, 10% glycerol, 25 mM β-glycerophosphate, 50 mM NaF, 2 mM Na_3_VO_4_ and Complete proteases inhibitors (Roche Diagnostics GmbH, Mannheim, Germany). Equal amounts of protein (30 µg) were fractionated by SDS-PAGE and transferred to nitrocellulose. Membranes were incubated with primary antibodies followed by appropriate IRDye-conjugated secondary antibodies, and developed and quantified using LI-COR (Odyssey). HEK-293-4.1B lysate was prepared from HEK293 cells stably expressing the rat 4.1B isoform initially identified by Ohara et al. (2000) [14].

### Immunofluorescence and quantitative studies

Immunostaining of teased fibers of sciatic nerves or cryostat sections of dorsal spinal roots (10 µm) was performed as previously described [59]. For detection of protein 4.1B, βII spectrin, and neurofilaments in sciatic nerves, tissues were fixed in paraformaldhehyde 4% for 30 minutes at room temperature and teased fibers were post-fixed with methanol/acetone 50/50 (volume/volume) for 20 minutes at −20°C. *In toto* immunostaining of neuromuscular junctions was performed as previously reported [60]. Images were acquired at the *Institut du Fer à Moulin* Cell and Tissue Imaging Facility, using a Leica SP2 confocal laser scanning microscope or an epifluorescence microscope Leica DM6000 microscope equipped with a CCD camera (Leica, Mannheim, Germany). Distances between nodes or incisures and axon diameters were measured using MetaMorph software (Universal Imaging Corporation, Downington, PA).

### Electrophysiology

#### Nerve electrophysiology

Sciatic nerves were quickly dissected, transferred into artificial cerebrospinal fluid (ACSF) and recording of CAPs was performed at 22°C and 35°C in a three-compartment recording chamber as previously described [61]. Drugs were applied in the central compartment of the chamber. For recruitment analysis, nerves were stimulated at increasing intensities and the CAP amplitudes were plotted as a function of the stimulation intensity. For refractory period analysis, two successive stimuli were applied at different intervals, and the amplitude of the second CAP was measured and plotted as a function of the delay between the two stimuli.

#### Compound muscle action potential (CMAP) recordings

Diaphragms with the attached phrenic nerves and ribs were quickly dissected-out, pinned down in a recording chamber and perfused with oxygenated Rees's solution as previously described [62]. The phrenic nerves were stimulated with a bipolar electrode while the nerve CAPs were recorded from the cut end with a suction electrode. The CMAPs were recorded simultaneously with a surface electrode pressed gently on the diaphragm. Diaphragms were continuously superfused with oxygenated Rees's solution in which drugs were applied. Recordings were made few millimeters away from the nerve endplates, and at the same location in all experiments. Spontaneous and repetitive activities were analyzed using Clampfit 9.2 (Axon Instruments).

### Behavioral analysis

Mice were group-housed with *ad libitum* access to food and water and a 12 hours − 12 hours light-dark cycle (light phase onset at 7 a.m.). Most experiments and initial quantitative analyses were performed in a ‘blind’ fashion, i.e., the experimenter had no knowledge of the genotype of the mice tested and analyzed.

#### Footprint pattern analysis (4- and 12-month-old mice)

The gait analysis method was adapted from de Medinaceli et al. (1982) [63] and Ozmen et al. (2002) [64]. The footprints were obtained by dipping mice hind paws into ink before they walked on white paper down a confined walkway 4.5-cm wide and 100-cm long with 10-cm high walls and a dark shelter at the end. The footprints patterns generated were scored for 2 parameters: step length and width (ImageJ software, NCBI). Step length was defined as the average distance of forward movement between at least three alternate steps. Gait width corresponded to the average lateral distance between opposite left and right steps. Alternation coefficient was calculated by the mean of the absolute value of 0.5 minus the ratio of right-left distance to right-right step distance for every left-right step pair.

#### Measurement of locomotor activity (4- to 6-month-old mice)

Spontaneous locomotor activity was measured using a circular corridor with four infrared beams placed at every 90° of the inferior part of the corridor (Imetronic, Pessac, France), in a low luminosity environment, avoiding stress. Ambulation (crossover of two inferior adjacent beams) was quantified in 5 minutes bins during 45 minutes with a computer software (Imetronic, Pessac, France), and cumulative counts were taken for data analysis.

#### Grip strength analysis (4- to 6-month-old mice)

Forelimb grip strength was measured using a Grip Strength Meter (Bioseb, Chaville, France) as recommended by the manufacturer. Briefly, mice were held by the tail and allowed to grasp a grid with their forepaws. Once the mouse grasped the grid with both paws, it was pulled away from the grid until it released the bar. The digital meter displayed the level of tension (in newtons) exerted on the grid by the mouse.

#### Hot plate (4- to 5-month-old mice)

A standard hot plate (Bioseb, Chaville, France), adjusted to 52°C, was used to assess motor reactions in response to noxious stimuli. The mouse was confined on the plate by a Plexiglas cylinder (diameter 19 cm, height 26 cm). The latency to a hind paw response (licking or shaking) or jumping, whatever happened first, was taken as the nociceptive threshold.

### Electron microscopy and morphometry

Electron microscopy and morphometry were performed as previously described [56]. Fibers diameters, axonal diameters, and G-ratios, defined as ratios of axonal to fiber's diameter were measured automatically after segmentation, in more than 100 fibers. Five to ten nodes of Ranvier were examined in longitudinal sections of sciatic nerves from 4- and 13-month-old mice of each genotype (2 mice per group).

### Statistical Analysis

Data in figures and text are expressed as means±s.e.m. They were compared by Student's *t*-test, Mann-Whitney non-parametric test, 1-way ANOVA followed by Bonferroni's Multiple Comparison Test, 2-way ANOVA followed by Bonferonni post-tests or by 2-way RM-ANOVA test, Mantel-Cox test and Chi 2 test where appropriate, using GraphPad Prism software (GraphPad Software, Inc., La Jolla, CA, USA). A *P* value below 0.05 was considered to be significant.

## Supporting Information

Figure S1 **Generation of protein 4.1B mutant mice.** A three-lox recombination strategy [24] was used to generate mutant mice presenting a *protein 4.1B* allele deleted from the exon 3 of the *Dal-1* gene (*protein 4.1B^KO3^*). **A.** Generation of the *protein 4.1B^KO3^* allele. In a first step, A 17.0-kb *protein 4.1B* genomic clone including exons 2 and 3 was isolated from a mouse 129/Ola genomic library. A 9.5-kb *Nsi*I-*Not*I fragment of this clone was used for the construction of the *protein 4.1B^lox3^* targeting fragment. This fragment was subcloned in a modified pBR322 vector containing a *Nhe*I-*Sal*I polylinker from pBluescript KS II (Stratagene). A 1.0-kb *Apa*I-*Apa*I fragment containing *protein 4.1B* exon 3 was amplified by PCR using primers containing *loxP* sites (open triangles) and was inserted between 2 corresponding *Apa*I sites, flanking *protein 4.1B* exon 3 of the 9.5-kb fragment. A 2.9-kb *Nhe*I-*Xba*I fragment containing a floxed PGK*Hygromycin*/*GFP* cassette was inserted into a *Spe*I site 0.5-kb downstream of exon 3 in the same orientation of the *protein 4.1B* gene. The *Nsi*I-*Not*I *protein 4.1B^lox3^* targeting fragment was electroporated into ES cells line E14 subclone IB10 [65]. Electroporated cells were plated on mouse embryonic fibroblasts and selected with hygromycin B. Hygromycin-resistant cells were trypsinized and GFP expressing cells were isolated and then plated on 96-well microplates by flow cytometric analysis. Homologous recombinants were identified by long-range PCR analysis. Four of six analyzed clones showed a correct karyotype. In the four diploid ES cell clones, 5′ and 3′ homologous recombination was confirmed by Southern blot analysis using digestions and probes. In a second step, germline chimeras (*Protein 4.1B^flox3HygGFP/+^*) were generated by injection of *protein 4.1B* mutant ES cells into C57BL/6 blastocysts and crossed with FVB/N mice to produce outbred heterozygous offspring. The genotypes of all offspring were analyzed by PCR or Southern blot analysis on tail-tip DNA. To generate *protein 4.1B^KO3/+^* mice, *protein 4.1B^flox3HygGFP/+^* mice were crossed with *EIIACre* deletor mice [54]. In the deriving double transgenic offspring tail DNA the *protein 4.1B^KO3^* allele, and the *protein 4.1B* allele with two *loxP* sites flanking the exon 3 of the gene (*protein 4.1B^ flox3^* allele), were detected by PCR. Restriction sites used for screening ((S) SwaI, (Ns) NsiI, (B) BamHI), DNA fragments resulting from digestions (doubled-headed arrows) and hybridization probes (A, B and Hygro (black boxes)) are indicated. Also depicted are the PCR primers DAL1afor (5′-TTCATTTGGTGAGATCATGG-3′), DAL1arev (5′-CTACCCGAAGAAGTGACTGG -3′) and DAL1bfor (5′-TAAAGTACGCTTTGCTCTAC-3′) that detected the different alleles. **B.** Long range PCR analysis to identified homologuous recombinant cells clones using the primers DAL1a.for and DAL1a.rev (PCR product of the *protein 4.1B^flox3^* allele 3.9 kb). **C.** Southern blot analysis of restricted DNA from a recombinant cells clone resulting from digestion with SwaI or BamHI. Hybridation with probes A and B shows complete integration of the targeting fragment after homologous recombination. Probe Hygro shows the single integration of targeting fragments after homologous recombination. **D, E.** Genotyping of modified *protein 4.1B* gene by PCR. Genomic DNA from mice was amplified by PCR using the primers DAL1bfor and DAL1arev to detect the *protein 4.1B^flox3^* allele (D) and the primers DAL1afor and DAL1arev to detect the *protein4.1B^KO3^* allele (E). In (E) *protein 4.1B^KO3/+^* mice were intercrossed. One litter of 11 pups was genotyped by PCR as described above and 5 *protein 4.1B^+/+^* mice, 3 *protein 4.1B^KO3/+^* mice, and 3 *protein 4.1B^KO3/KO3^* mice were found, demonstrating that protein 4.1B is not required for mouse development. w: *protein 4.1B^+/+^* mice; h: *protein 4.1B^KO3/+^* mice; H: *protein 4.1B^KO3/KO3^* mice (referred in the manuscript as “4.1B KO mice”).
(TIF)

Figure S2 **Protein 4.1B isoforms expression in rat PNS and CNS.** **A.** Schematic representation of the domain structure of rat 4.1B (KIAA0987) [4] : FERM, four point one-ezrin-radixin-moesin domain; FA, FERM-adjacent domain; SAB, spectrin-actin-binding domain; CTD, carboxy-terminal domain; U1, U2, U3, domains distinct in each 4.1 protein. The amino acid residues bordering the different domains are indicated. The position of the protein fragment (residues P612-L804) used to raised the antibodies (4.1B pAb) is indicated by a doubled-headed arrow. N, amino-terminal part of the protein; C, carboxy-terminal part of the protein. **B.** Immunoblots performed on lysates of rat peripheral nerves (sciatic, brachial and trigeminal nerves) and CNS tissues (brain, spinal cord, optic nerve). **C.** Immunoblots performed on lysates of HEK293 cells expressing the rat 4.1B isoforms initially identified by Ohara et al. (2000) [14], and lysates of rat brain and sciatic nerve. Different isoforms of 4.1B are preferentially expressed in the peripheral and central nervous tissues, respectively. Main isoforms, arrows.
(TIF)

Figure S3 **Survival of heterozygous and homozygous 4.1B mutant mice.** Survival curves of mice over a period of 18 months. Significant increased lethality of homozygous 4.1B mutant mice (-/-, *n = *27, 9 females and 18 males) after 6 months was observed compared to heterozygous 4.1B mutant mice (+/-, *n = *17, 10 females and 7 males). Dead mice were mainly diagnosed for kidney, liver and spleen inflammation, or begnin tumors (ovary, pituitary, mammary gland, lung) (data not shown). Statistical test, Mantel-Cox test, Chi 2 = 3.974, *p* = 0.0462.
(TIF)

Figure S4 **Axonal and myelin proteins levels are not altered in sciatic nerves from 4.1B KO mice. A, C.** Immunoblots of P0, myelin basic protein (MBP) (A), neurofilament subunits NF-L, NF-M, NF-H, phosphorylated NF-H (NF-H-Ph), actin (C) and neuronal class III β-tubulin (Tubulin, A, C) in sciatic nerves from wild type (+/+), 4.1B heterozygous (+/-) and homozygous (-/-) KO mice. **B, D.** Quantification of proteins expression normalized to β-tubulin signal (three mice for each genotype). Statistical analysis, 1-way ANOVA followed by Bonferroni's multiple comparison test. No significant change was observed between the different genotypes.
(TIF)

Figure S5 **CAPs from sciatic nerves of 5-month-old WT and 4.1B KO mice recorded at 35°C. A.** Representative CAPs recorded from WT and KO sciatic nerves. Superposition (Grey) of the WT CAPs with the KO CAPs shows that conduction is not affected in mutant animals. The places used to calculate V_1/2_ and V_max_ are indicated (grey arrowheads). **B.** Normal refractory period of the CAPs from WT and KO mice sciatic nerves. **C.** Similar CAPs recruitment in sciatic nerves of WT and KO mice. WT, *n = *9; KO, *n = *10.
(TIF)

Figure S6 **Neuromuscular hyperactivity in 4.1B KO mice during development.** Evoked (A) and spontaneous (B) neuromuscular hyperactivity was quantified during development (2 to 25 weeks) in the absence and in the presence of the potassium channel blocker TEA (5 mM). Note that hyperexcitability is transient during development and that TEA exacerbates both repetitive and spontaneous activities.
(TIF)
