# Supplementary figures and images for: Protein 4.1B Contributes to the Organization of Peripheral Myelinated Axons
Source: PLoS One. 2011 Sep 26;6(9):e25043. doi: 10.1371/journal.pone.0025043 (PMC3180372; doi:10.1371/journal.pone.0025043)

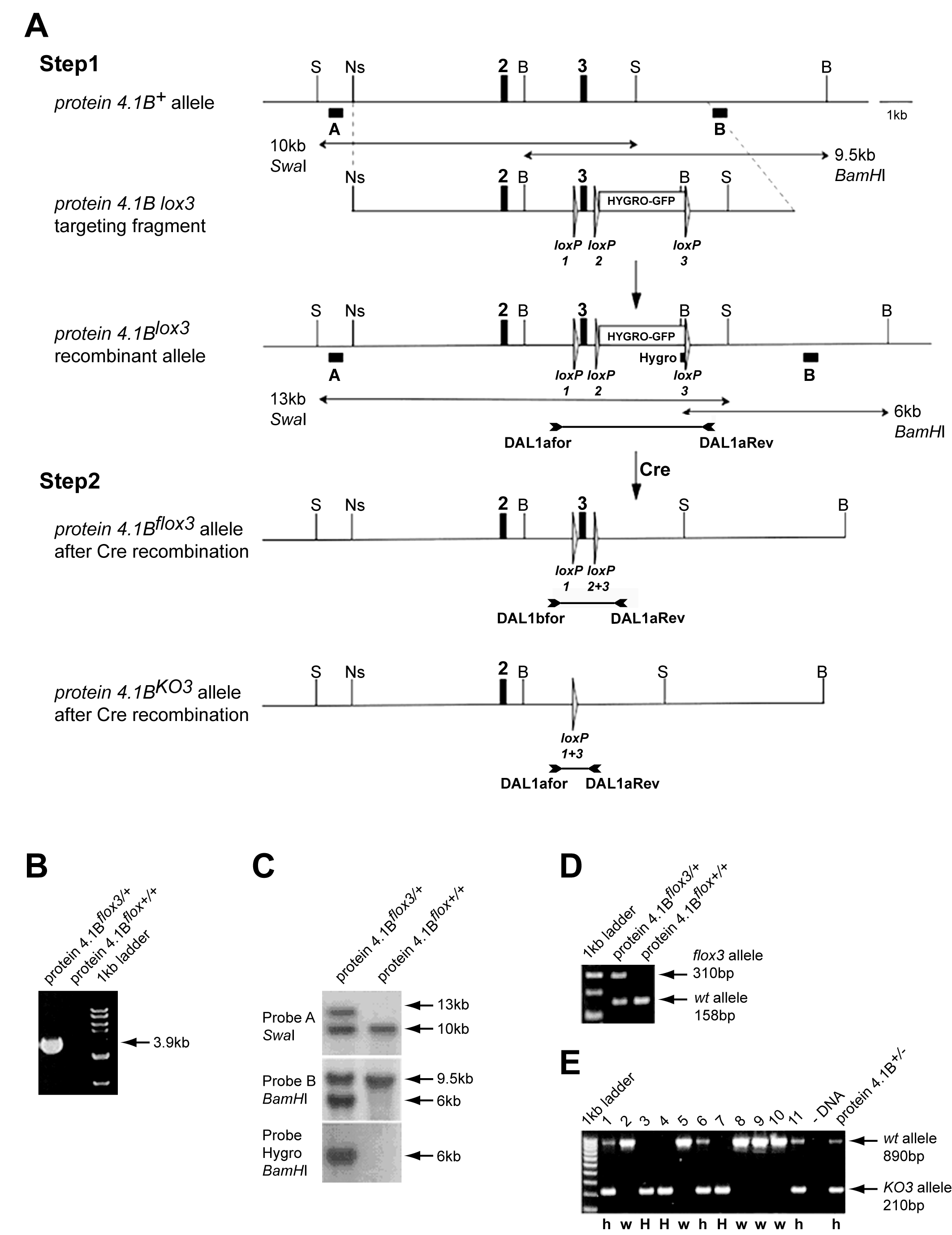

Supplement: Figure S1 — Generation of protein 4.1B mutant mice. A three-lox recombination strategy [24] was used to generate mutant mice presenting a protein 4.1B allele deleted from the exon 3 of the Dal-1 gene (protein 4.1BKO3). A. Generation of the protein 4.1BKO3 allele. In a first step, A 17.0-kb protein 4.1B genomic clone including exons 2 and 3 was isolated from a mouse 129/Ola genomic library. A 9.5-kb NsiI-NotI fragment of this clone was used for the construction of the protein 4.1Blox3 targeting fragment. This fragment was subcloned in a modified pBR322 vector containing a NheI-SalI polylinker from pBluescript KS II (Stratagene). A 1.0-kb ApaI-ApaI fragment containing protein 4.1B exon 3 was amplified by PCR using primers containing loxP sites (open triangles) and was inserted between 2 corresponding ApaI sites, flanking protein 4.1B exon 3 of the 9.5-kb fragment. A 2.9-kb NheI-XbaI fragment containing a floxed PGKHygromycin/GFP cassette was inserted into a SpeI site 0.5-kb downstream of exon 3 in the same orientation of the protein 4.1B gene. The NsiI-NotI protein 4.1Blox3 targeting fragment was electroporated into ES cells line E14 subclone IB10 [65]. Electroporated cells were plated on mouse embryonic fibroblasts and selected with hygromycin B. Hygromycin-resistant cells were trypsinized and GFP expressing cells were isolated and then plated on 96-well microplates by flow cytometric analysis. Homologous recombinants were identified by long-range PCR analysis. Four of six analyzed clones showed a correct karyotype. In the four diploid ES cell clones, 5′ and 3′ homologous recombination was confirmed by Southern blot analysis using digestions and probes. In a second step, germline chimeras (Protein 4.1Bflox3HygGFP/+) were generated by injection of protein 4.1B mutant ES cells into C57BL/6 blastocysts and crossed with FVB/N mice to produce outbred heterozygous offspring. The genotypes of all offspring were analyzed by PCR or Southern blot analysis on tail-tip DNA. To generate prot [file pone.0025043.s001.tif]

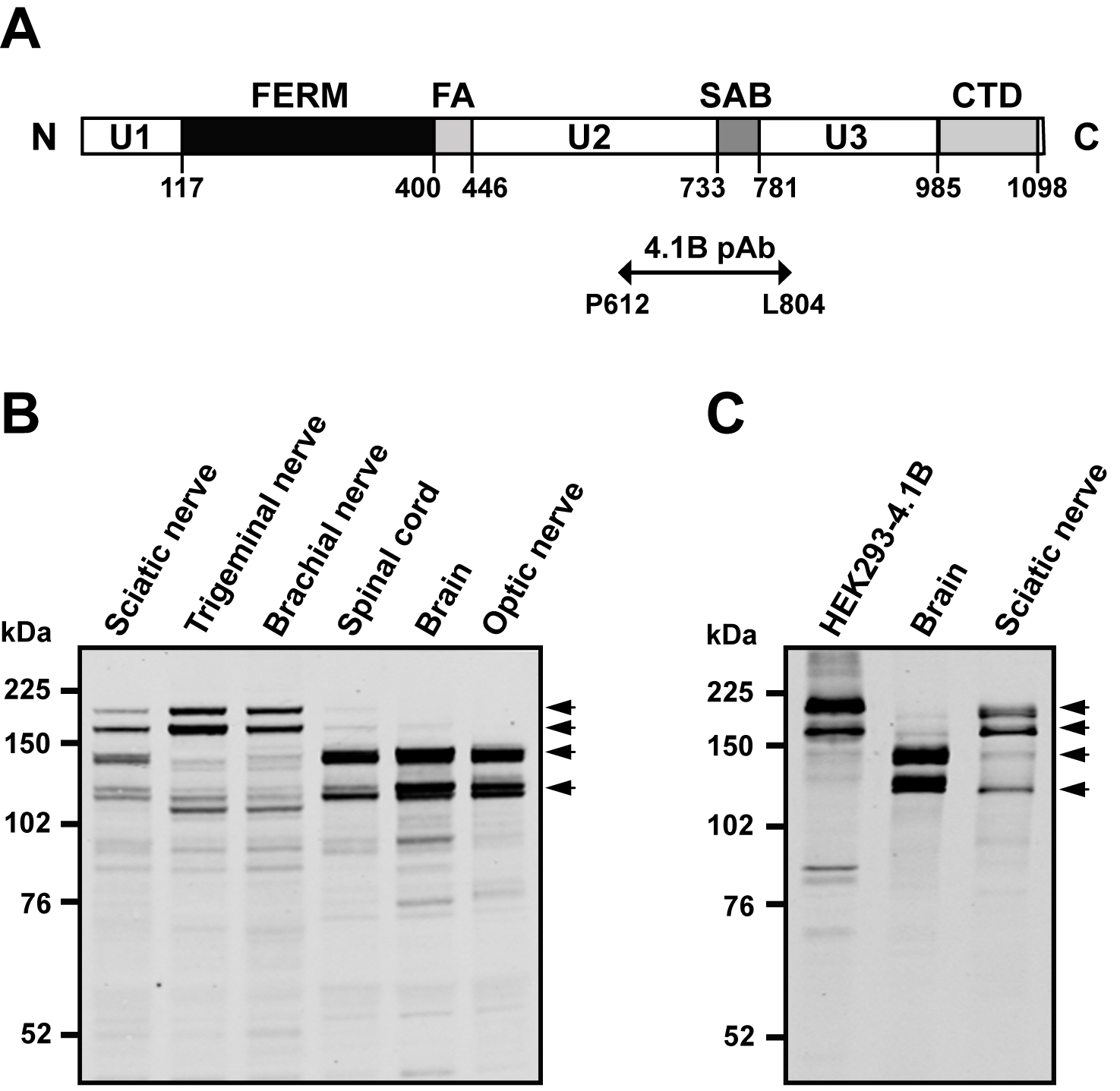

Supplement: Figure S2 — Protein 4.1B isoforms expression in rat PNS and CNS. A. Schematic representation of the domain structure of rat 4.1B (KIAA0987) [4] : FERM, four point one-ezrin-radixin-moesin domain; FA, FERM-adjacent domain; SAB, spectrin-actin-binding domain; CTD, carboxy-terminal domain; U1, U2, U3, domains distinct in each 4.1 protein. The amino acid residues bordering the different domains are indicated. The position of the protein fragment (residues P612-L804) used to raised the antibodies (4.1B pAb) is indicated by a doubled-headed arrow. N, amino-terminal part of the protein; C, carboxy-terminal part of the protein. B. Immunoblots performed on lysates of rat peripheral nerves (sciatic, brachial and trigeminal nerves) and CNS tissues (brain, spinal cord, optic nerve). C. Immunoblots performed on lysates of HEK293 cells expressing the rat 4.1B isoforms initially identified by Ohara et al. (2000) [14], and lysates of rat brain and sciatic nerve. Different isoforms of 4.1B are preferentially expressed in the peripheral and central nervous tissues, respectively. Main isoforms, arrows. (TIF) [file pone.0025043.s002.tif]

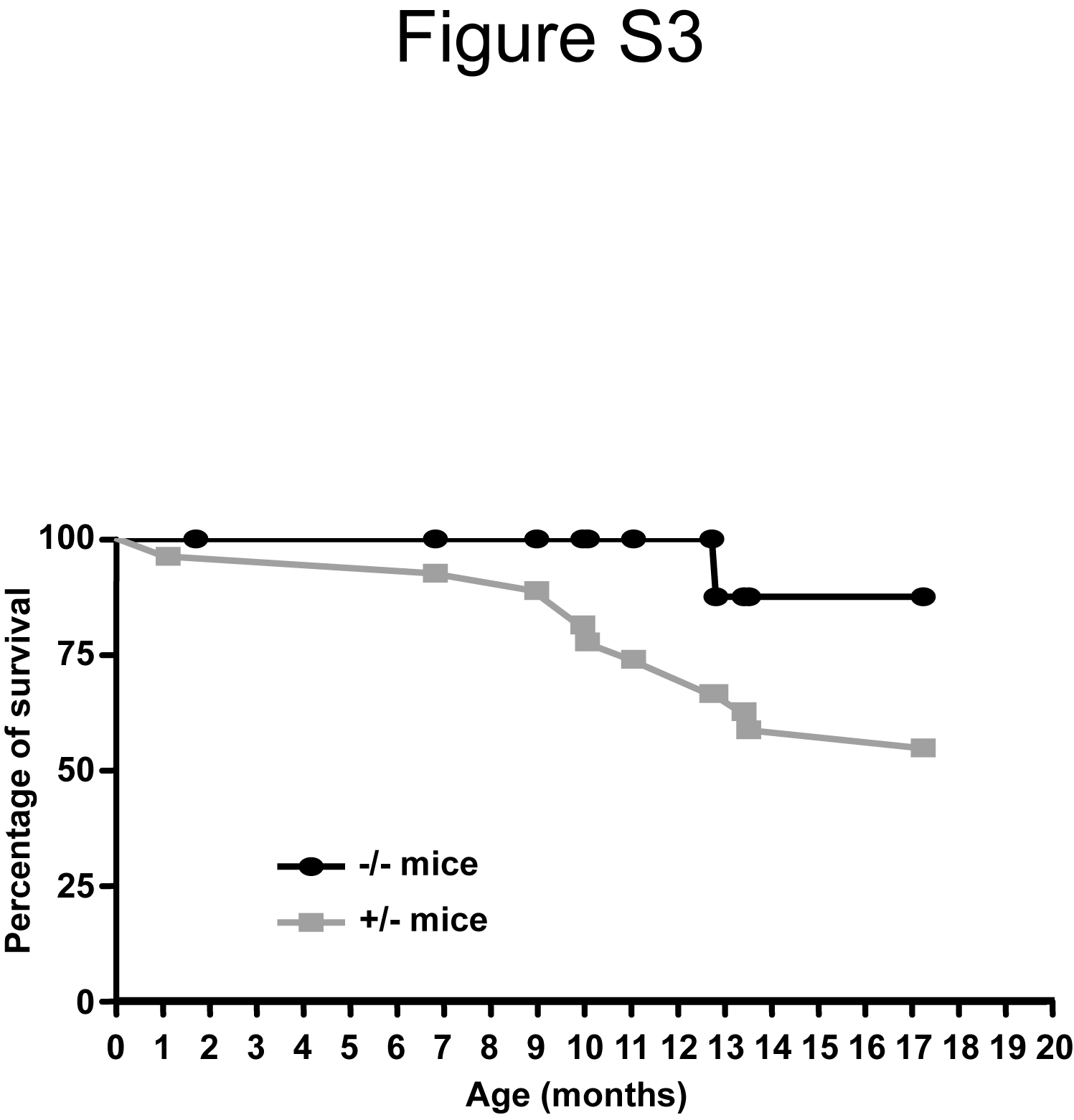

Supplement: Figure S3 — Survival of heterozygous and homozygous 4.1B mutant mice. Survival curves of mice over a period of 18 months. Significant increased lethality of homozygous 4.1B mutant mice (-/-, n = 27, 9 females and 18 males) after 6 months was observed compared to heterozygous 4.1B mutant mice (+/-, n = 17, 10 females and 7 males). Dead mice were mainly diagnosed for kidney, liver and spleen inflammation, or begnin tumors (ovary, pituitary, mammary gland, lung) (data not shown). Statistical test, Mantel-Cox test, Chi 2 = 3.974, p = 0.0462. (TIF) [file pone.0025043.s003.tif]

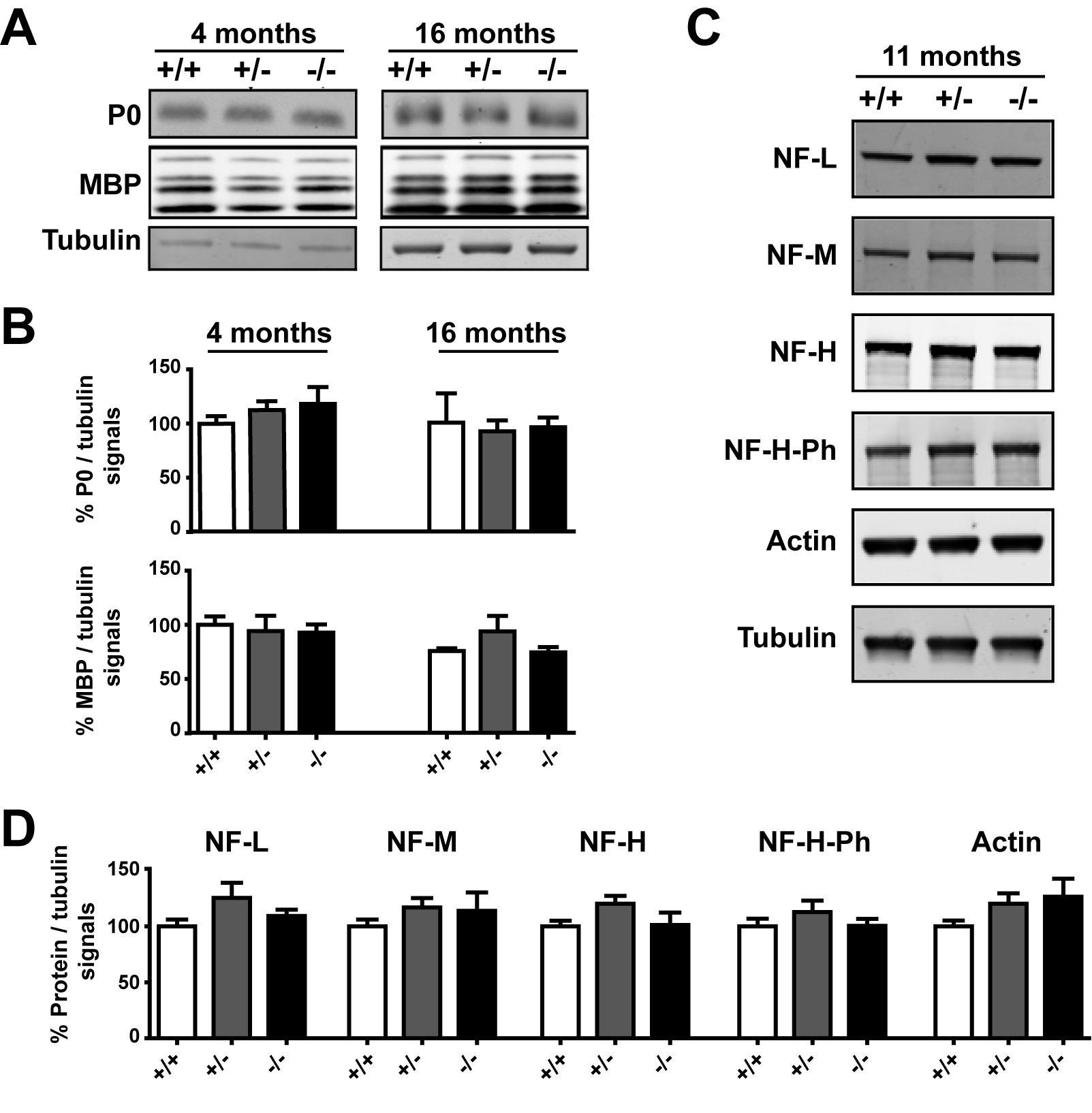

Supplement: Figure S4 — Axonal and myelin proteins levels are not altered in sciatic nerves from 4.1B KO mice. A, C. Immunoblots of P0, myelin basic protein (MBP) (A), neurofilament subunits NF-L, NF-M, NF-H, phosphorylated NF-H (NF-H-Ph), actin (C) and neuronal class III β-tubulin (Tubulin, A, C) in sciatic nerves from wild type (+/+), 4.1B heterozygous (+/-) and homozygous (-/-) KO mice. B, D. Quantification of proteins expression normalized to β-tubulin signal (three mice for each genotype). Statistical analysis, 1-way ANOVA followed by Bonferroni's multiple comparison test. No significant change was observed between the different genotypes. (TIF) [file pone.0025043.s004.tif]

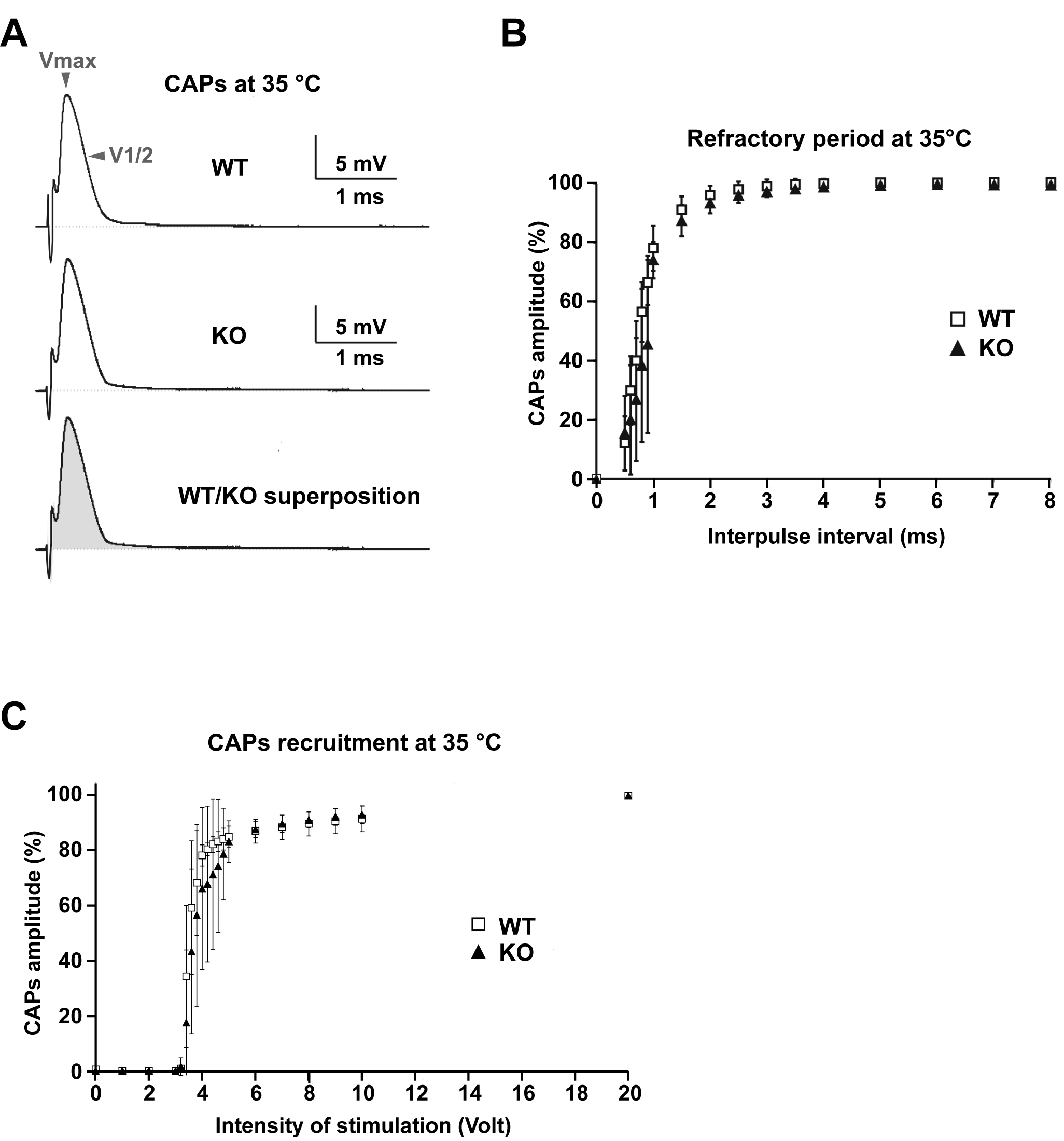

Supplement: Figure S5 — CAPs from sciatic nerves of 5-month-old WT and 4.1B KO mice recorded at 35°C. A. Representative CAPs recorded from WT and KO sciatic nerves. Superposition (Grey) of the WT CAPs with the KO CAPs shows that conduction is not affected in mutant animals. The places used to calculate V1/2 and Vmax are indicated (grey arrowheads). B. Normal refractory period of the CAPs from WT and KO mice sciatic nerves. C. Similar CAPs recruitment in sciatic nerves of WT and KO mice. WT, n = 9; KO, n = 10. (TIF) [file pone.0025043.s005.tif]

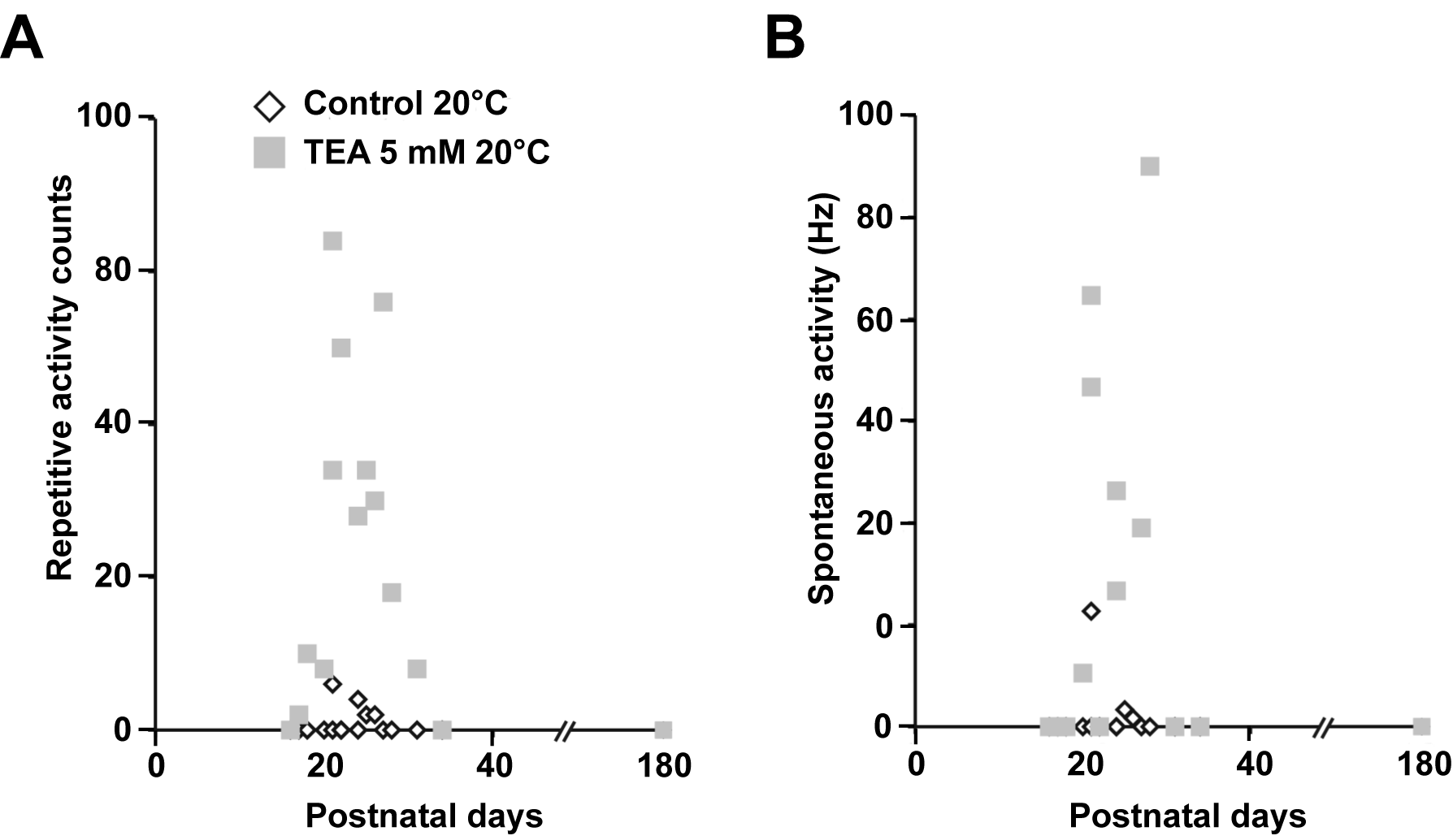

Supplement: Figure S6 — Neuromuscular hyperactivity in 4.1B KO mice during development. Evoked (A) and spontaneous (B) neuromuscular hyperactivity was quantified during development (2 to 25 weeks) in the absence and in the presence of the potassium channel blocker TEA (5 mM). Note that hyperexcitability is transient during development and that TEA exacerbates both repetitive and spontaneous activities. (TIF) [file pone.0025043.s006.tif]
